# CytoCopasi: a chemical systems biology target and drug discovery visual data analytics platform

**DOI:** 10.1093/bioinformatics/btad745

**Published:** 2023-12-09

**Authors:** Hikmet Emre Kaya, Kevin J Naidoo

**Affiliations:** Department of Chemistry, Scientific Computing Research Unit, PD Hahn Building, University of Cape Town, Rondebosch 7701, South Africa; Department of Chemistry, Scientific Computing Research Unit, PD Hahn Building, University of Cape Town, Rondebosch 7701, South Africa

## Abstract

**Motivation:**

Target discovery and drug evaluation for diseases with complex mechanisms call for a streamlined chemical systems analysis platform. Currently available tools lack the emphasis on reaction kinetics, access to relevant databases, and algorithms to visualize perturbations on a chemical scale providing quantitative details as well streamlined visual data analytics functionality.

**Results:**

CytoCopasi, a Maven-based application for Cytoscape that combines the chemical systems analysis features of COPASI with the visualization and database access tools of Cytoscape and its plugin applications has been developed. The diverse functionality of CytoCopasi through ab initio model construction, model construction via pathway and parameter databases KEGG and BRENDA is presented. The comparative systems biology visualization analysis toolset is illustrated through a drug competence study on the cancerous RAF/MEK/ERK pathway.

**Availability and implementation:**

The COPASI files, simulation data, native libraries, and the manual are available on https://github.com/scientificomputing/CytoCopasi.

## 1 Introduction

Data visualization methods hold the key to effective analysis of complex large systems that lay at the heart of understanding the genotype–phenotype relationship. Genome-scale metabolic models that contain all the known biochemical reactions within cells provide a mechanistic understanding and link between the genotype and phenotype. Reducing biological data to networks of interacting proteins and metabolites makes possible the piecing together of biochemical sequences and events that underlie cellular function. Presenting this metadata on a platform through interactive graphics can greatly enable scientific discovery. A platform used for the mathematical modeling of biochemical ecosystems requires user-friendly tools able to construct, edit, compute, and visualize complex reactions interacting with metabolites. To compose the metadata from which biological models are derived there is a fundamental requirement to extract complex pathway maps and reaction parameters in a high throughput schema. Consequently, large databases must necessarily be interfaced with the platform to allow workflows for model construction. These models function to test and refine the networks that lead to a molecular mechanistic understanding of biological phenomena.

Biochemical reaction networks are often modeled using constraint-based methods (Bordbar *et al.* 2014) such as Flux Balance Analysis (FBA) (Savinell and Palsson 1992, Varma *et al.* 1993) that lends itself to large genome-scale metabolic network building. In the FBA scheme, linear stoichiometric equations for each molecular compound are drawn up in rows and are subject to reactions depicted in columns collectively making up a matrix (Price and Shmulevich 2007). These methods are based on mass balance across a metabolic network and are ideal for the integration of different omics at the genome length scale that improves the model’s predictive performance for large systems, such as cellular growth rate or the production rate of a metabolite, under different metabolic conditions (Vital-Lopez *et al.* 2013). However, advancement in a personalized approach to disease prediction and care requires dynamic models able to predict metabolic response to altered reaction networks and measure metabolite concentration due to inhibition reaction rates.

In place of making the steady-state assumptions to build static models using linear equation tool sets, dynamic models are built from ordinary differential equations (ODEs) that include kinetic rate constants. These dynamic models are well positioned for use in rational drug design and the simulation of induced molecular perturbations such as from diseased states. Although it is noted that a major drawback of the kinetic modeling is the large data dependence from kinetic parameters for each reaction in the network (Price and Shmulevich 2007). However, a systems approach to target discovery and evaluation of drug efficacy is practically possible if coupled with appropriate databases and simple-to-use workflows for nonexperts. Although challenging, gathering model visualization, editing, and analysis tools on a single platform and linking them to various biological repositories can accelerate hypothesis generation in systems biology studies. Such a comprehensive platform is integral to the automation of chemical systems biology analysis and the mitigation of human errors. However, current reaction network modeling tools lack the complete bouquet of features.

Popular systems biology tools, such as Complex Pathway Simulator (COPASI) (Hoops *et al.* 2006) and CellDesigner (Funahashi *et al.* 2006), despite a plethora of simulation and analysis tools, do not offer a seamless workflow for comparative systems biology calculations. Visualizing the effects of model perturbations, such as enzyme mutation and drug administration, directly on the uploaded models is not easily done. Previous attempts use manually created data sheets, heatmaps, and abstract network images to present results, potentially leading to data discrepancies (Mishra *et al.* 2018). Current tools have shortcomings such as COPASI’s lack of access to any pathway or enzyme kinetics database without the knowledge of specific model IDs or URLs through a browser. Moreover, CellDesigner’s database access, which is limited to BioModels (Le Novère *et al.* 2006) for importing models and System for the Analysis of Biochemical Pathways—Reaction Kinetics (SABIO-RK) (Wittig *et al.* 2012) for extracting kinetic laws, limits the scope of systems studies to previously curated kinetic models. In the absence of such models, integration with larger databases, such as the Kyoto Encyclopedia of Genes and Genomes (KEGG) (Kanehisa and Goto 2000) and the Braunschweig Enzyme Database (BRENDA) (Schomburg *et al.* 2002) enable construction of large custom models. Although there is the caveat that data acquisition from web interfaces into a systems biology platform can introduce human-errors into the process.

Overall, while these tools have excellent track records in stand-alone modeling and simulation, integrating them into a single platform able to create large study pipelines is presently absent. Therefore, the outstanding challenge to deploy biology tools onto software platforms that integrate multiple analytic functionalities and databases, remains.

Cytoscape (Shannon *et al.* 2003), a Java-based application for visualizing and analyzing molecular interaction networks, serves as an umbrella for a growing family of applications. These applications are freely available and mostly open source, with a well-established app development community. At the time of writing, the Cytoscape App Store (Lotia *et al.* 2013) has a repository of 373 apps in many categories, including network generation, clustering, online data import, enrichment analysis, and systems biology. However, Cytoscape in its design and user base has been intentionally aimed at genome-wide modeling using statistical methods applied to gene sequencing and expression data. None the less Cytoscape-based applications can enable modeling and simulations of cellular signaling pathways based on topological information, protein activity level, or gene expression. Applications such as PathInsight (Venkat 2017) have a simplified and qualitative approach to model perturbation, which can be used to predict the downstream effects of mutations and drug interventions. Quantitative methods applications include Pathway Signal Flow Calculator (PSFC) (Nersisyan *et al.* 2015) that computes the signal propagation from model inputs to the outputs based on gene expression and protein activity.

Central to cellular maps are enzyme kinetic systems driven biological processes involving post-translational modifications (PTMs) principally, glycosylation, phosphorylation, and methylation. The assumption that gene expression directly translates into protein activity and function does not account for PTMs, specifically the enzymatically constructed glycoconjugates and the interaction of glycans (complex carbohydrates) with proteins (Neelamegham and Liu 2011). The Cytoscape platform and associated apps are limited to genomics and proteomics analytics and are disconnected from modeling reaction kinetics in complex biological networks. Fine-grained models that include biochemical metabolic networks and chemical interventions such as small molecule enzyme inhibition are absent from platforms such as Cytoscape. Consequently, the overall goal of precision medicine modeling cannot be achieved through seamless pipelines connecting genome-wide models, protein-level cellular networks, and fine-grained biochemical reactions. To make this possible, the integration of kinetic models into platforms such as Cytoscape is necessary.

Cytoscape with its network modeling and visual analytics apps is an ideal platform to host the COPASI package. The objective is to undertake reaction rate calculations for biochemical networks and graphically analyze dynamic changes due to altered reaction rates within the network. There is the caveat that several module modifications are needed to adapt both the Cytoscape platform and the COPASI package to facilitate the integration. Here, we introduce the Cytoscape application CytoCopasi that enables the visualization, simulation, and analysis of complex reaction networks via COPASI.

## 2 Materials and methods

### 2.1 CytoCopasi overview

CytoCopasi implements the Java bindings of Complex Pathway Simulator (COPASI) (Hoops *et al.* 2006) for modeling and simulating biochemical reaction networks in Cytoscape (Shannon *et al.* 2003). It is the first Cytoscape application that performs chemical systems biology simulations based on mass action law and ODE-based deterministic time series. Further, it facilitates fast connection to databases to acquire enzyme functional parameters and pathway maps.

**Figure 1.**
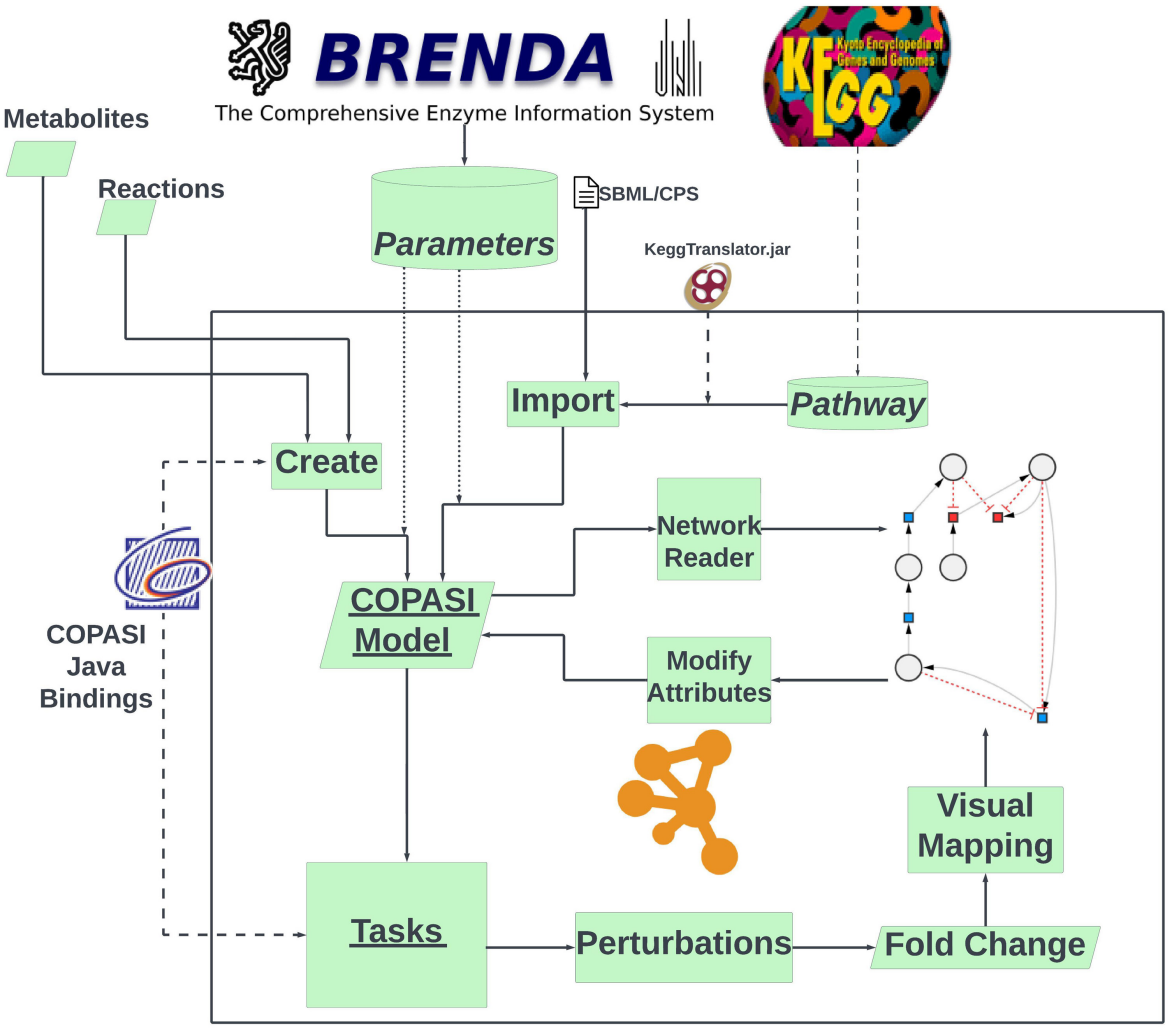
Overview of CytoCopasi showing a biochemical systems biology built from COPASI that merge visualization, curation, and analysis components on the Cytoscape platform.

The modeling, visualization, and simulation components of CytoCopasi are interconnected (Fig. 1) such that users can manually create biochemical networks by adding metabolites and reactions and assigning attributes to these model elements through network editing dialogs. Metabolite attributes include initial concentration and the metabolite state (i.e. whether its concentration is fixed or dependent on the reaction rate law), while reaction attributes include reaction equations, reversibility, rate law, and the associated parameters.

A rate law can either be selected from the COPASI function database or defined by the user. User-entered chemical equations result in a CytoCopasi-produced list of suitable rate law functions that are based on the number of reactants and products. To overcome the large kinetic data requirements, functional parameters for enzymatic reactions can be queried and retrieved from the Braunschweig Enzyme Database (BRENDA) (Schomburg *et al.* 2002) via Simple Object Access Protocol (SOAP) (Box *et al.* 2000) access.

CytoCopasi supports Systems Biology Markup Language (SBML) import and export functionalities enabling COPASI's SBML Java bindings (Hucka *et al.* 2003). Users can either locally upload SBML files or download pathway maps from the Kyoto Encyclopedia of Genes and Genomes (KEGG) (Kanehisa and Goto 2000). CytoCopasi uses a modified version of the Cytoscape application CyKEGGParser’s algorithm (Nersisyan *et al.* 2014) to download organism-specific pathways through KEGG's Rest API. Similar to CyKEGGParser, it calls the third-party Java application KEGGTranslator (Wrzodek *et al.* 2011) for converting KEGG Markup Language (KGML) files to SBML. However, CytoCopasi modifies the original KGML file ahead of conversion to make it compatible with the biochemical reaction network representation.

A major CytoCopasi feature is the ease of COPASI model representation as concise and informative networks in Cytoscape's network space. CytoCopasi networks are defined as combinations of metabolite nodes and reaction nodes, where edges connecting the nodes depict the direction of the network. In practice, every CytoCopasi model created or imported is built from an empty Cytoscape network accompanied by empty node and edge tables. A CyNode object, corresponding to an empty Cytoscape node, is then initiated for every element (i.e. metabolite or reaction) within the model. Properties of that element (either specified by the user or read from an imported file) are parsed into a series of attributes that populate cells in the node table row, whereby the cell entries are assigned to the Cytoscape node. The information on reactants and products is then used to create edges that connect metabolite nodes to the relevant reaction nodes.

CytoCopasi uses a simplified version of the visual style template of the Cytoscape application CySBML (König *et al.* 2012) to customize networks by displaying metabolites with circular nodes and reactions in square-shaped nodes. Reaction nodes are colored blue or red depending on whether the reaction is reversible or irreversible, respectively. The edge shape is determined by the interaction type. In the case that a metabolite acts as an inhibitor, the corresponding edge is represented as a red, dash line, and the default arrow is replaced with a T-shape. In addition to the visual style template used to construct network views, CytoCopasi offers versatility to further customize the visual properties and network layout.

A user-friendly dashboard for viewing model element details keeps the main network diagram active for editing model element details by double-clicking on nodes to foreground the requested data display. Modifications in node attributes are passed on to corresponding COPASI model element objects and autosaved.

Once a network is fully constructed, the concentration and flux dynamics can be computed via COPASI’s deterministic ODE simulator. Mechanisms of disease and drug action can then be explored using ODE simulations and steady-state kinetics. The complete workflow for the manual creation of and time course simulation of a model was illustrated in Supplementary Material S1, using a small-scale system by Fuentes *et al.* (2005) as an example.

In addition, CytoCopasi leverages COPASI’s simulation tasks to create a streamlined workflow for direct visual network comparison between the biological states of a system. On-the-fly experiments of perturbation effects across the entire network can lead to the detection of drug target areas.

### 2.2 Database integration

The connection between CytoCopasi and databases allows streamlined development of complex reaction networks without previously curated quantitative models. Essential data extraction, from two platforms via their web protocols are possible. Firstly, KEGG serves to generate a network topology with clearly defined metabolites and reactions. Following this, the reaction data can be populated through user-generated experiments or scanned in from previously reported work. At present, populating reaction kinetic parameters remains a manual task, as there are often multiple options for each reaction. However, this task is simplified through CytoCopasi’s connection with the enzyme functional database BRENDA, which displays the query results directly in a Cytoscape session.

#### 2.2.1 Extracting pathways from the KEGG database

CytoCopasi’s KEGG import feature is based on the Cytoscape App CyKEGGParser (Nersisyan *et al.* 2014), which uses KEGG’s Representational state transfer-style application programming interface (REST API) to request and retrieve data entries.

Similar to CyKEGGParser, CytoCopasi uses the third-party application KeggTranslator (Wrzodek *et al.* 2011) to convert KGML to SBML format.

A KEGG-imported model in CytoCopasi contains the same attributes as those of a locally imported SBML model while retaining the attributes inherent to the KGML file, such as complete compound and enzyme names, compound structures in GIF format, and hyperlinks to the KEGG entry pages.

#### 2.2.2 Extracting kinetic parameters from the BRENDA database

In instances where reaction network models are not completely parameterized, as is the case for KEGG pathways or newly created networks, a manual inquiry for kinetics and parameters would be needed. This requires rapid access to databases able to concisely present query results. Although BRENDA is the most comprehensive functional database to date, finding the parameters of interest with specific experimental conditions is not a seamless operation.

CytoCopasi makes enzyme functional parameter queries more deliberate by accessing BRENDA’s web services through a Simple Object Access Protocol (SOAP). It retrieves all the matching entries and presents the results in a panel displaying experimental conditions (pH, temperature, etc.), as well as the hyperlink to the corresponding PubMed study. Thus, parameter search is narrowed down to a specific organism but kept versatile enough that the user can select the appropriate organism reaction parameter.

#### 2.2.3 Model creation through in-platform accessible databases

**Figure 2.**
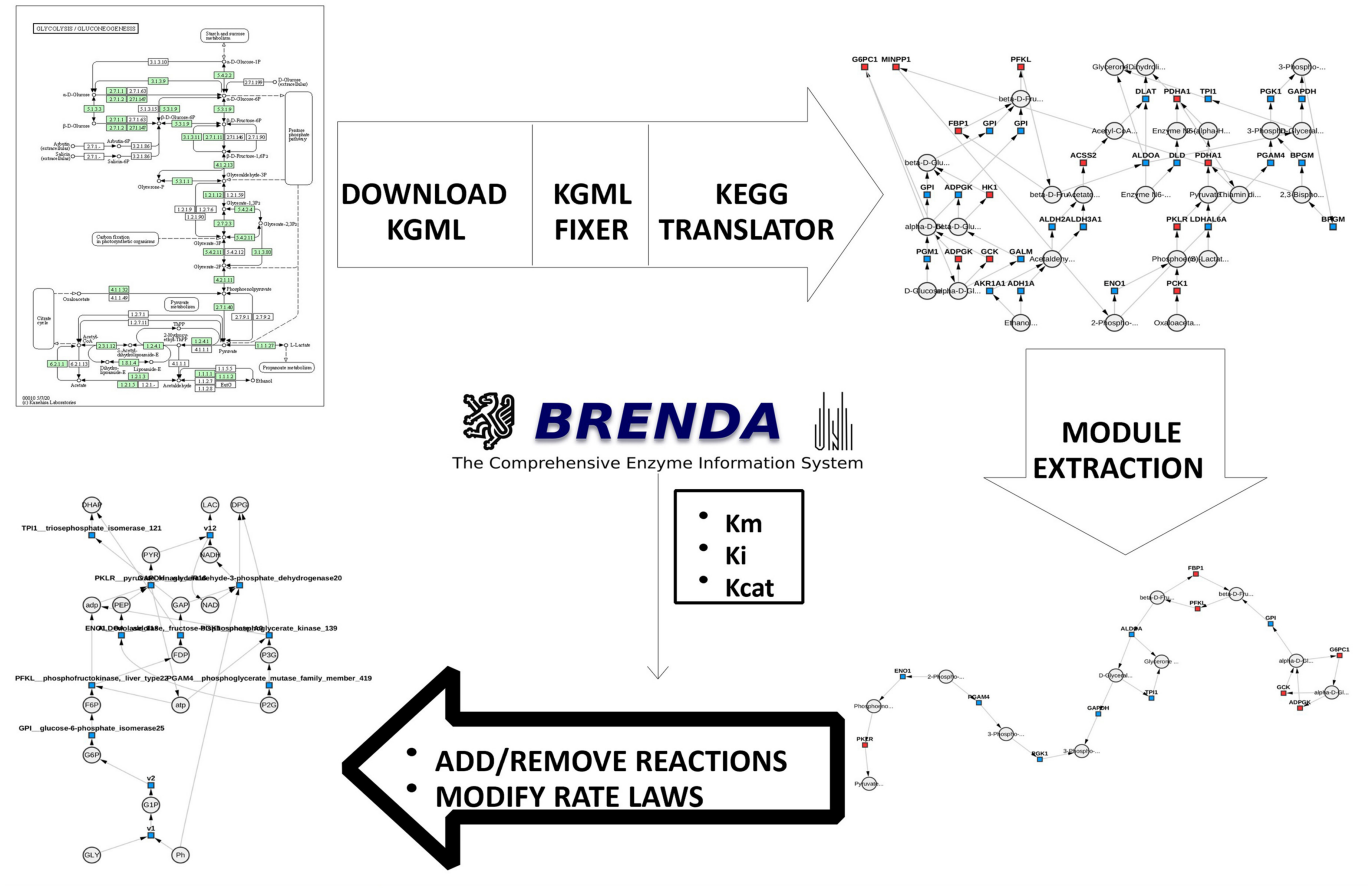
A workflow illustrating the generation of the skeletal muscle glycolysis model. The CytoCopasi extraction module is applied to the entire pathway in the KEGG Glycolysis Network (top right panel) to deliver the key reactions that are automatically customized and functional enzyme parameters obtained from BRENDA inserted to build the skeletal muscle glycolysis model (bottom left panel).

The functionality of KEGG and BRENDA in model construction is demonstrated by replicating the glycolysis model for skeletal muscle by Lambeth and Kushmerick (2002) (Fig. 2). The model contains 18 metabolites and 12 reactions and commences with the conversion of glycogen to D-Glucose 1-phosphate, traversing the KEGG Glycolysis pathway (Id: hsa00010) until the conversion of pyruvate to lactate.

While many KEGG pathways are made up of more than 30 reactions, they contain modules, subnetworks for specific groups of metabolites and enzymes that are of stand-alone biological importance. Upon importing a full KEGG pathway, CytoCopasi can extract a user-selected module as a new network.

KEGG’s Glycolysis/Glucogenesis network contains four modules: Glycolysis (Embden-Meyerhof pathway), Glycolysis—core module involving three-carbon compounds, Gluconeogenesis, and Pyruvate oxidation. Among the four modules, Glycolysis (Embden-Meyerhof pathway) (module ID: M0001) shows the greatest overlap with the model, hence the choice of the subnetwork. The steps of replicating the original model starting with the full KEGG Glycolysis Pathway are detailed in Supplementary Material S2 with the workflow illustrated here (Fig. 2).

The kinetic parameters on BRENDA were used to populate rate laws and parameters for each reaction. It is important to note that parameters exhibit variations across different literature entries due to variations in experimental conditions.

A common challenge in model building is the mixed use of enzyme reaction parameters taken from different organisms and experimental conditions. Here the principle of coherent model construction was adhered to with mainly rabbit-related data and inquiring after human-related data when rabbit kinetics were absent. Consequently, of the 12 reactions, five of them were parameterized entirely using BRENDA, with rabbit for four reactions and human for the remaining one. Three other reactions had 75%–80% of their parameters obtained from BRENDA, while two reactions only had half of the parameters available on the database. The remaining two reactions did not return any value for either species. The missing parameters were completed using the values from the original study. The parameters for this model for each reaction are described in Supplementary Material S2.

While CytoCopasi’s KEGG and BRENDA algorithms establish a robust connection to the database, limitations remain. For instance, because BRENDA congregates functional parameters of enzymes from a plethora of literature reports, the results show a great variety in terms of the range of values and experimental conditions. Selecting the most suitable parameter value during model construction necessitates user intervention, making this a bottleneck in a high throughput workflow. Future versions of CytoCopasi will replace manual parameter selection with semi-automated parameter extraction algorithms by passing the query results through user-defined filters to find the most consistent dataset for as many reactions as possible, thereby minimizing the number of reactions requiring manual parameter selection, ultimately achieving accelerated model construction for large systems comprising a large number of reactions.

### 2.3 Simulation and analysis

CytoCopasi performs deterministic time-course and steady state simulations through the Livermore Solver for Ordinary Differential Equations (LSODA or LSODAR) (Petzold 1983), the default deterministic simulation method in COPASI.

Comparative systems biology can be performed in two ways. The first approach involves comparing the simulation outputs of two COPASI models to illustrate the variations between their intersecting model elements in two different states. The other approach is based on COPASI’s parameter scan feature, which runs sequential ODE simulations on the same model while tweaking one or more parameters of interest. The effect of the perturbations on simulation output can be observed on the fly.

Comparison can be performed either between the time course-simulations or the steady state values of two models (given that a steady state can be found for both and that the models feature the same metabolites and reactions). The initial simulation output of a model (i.e. transient concentrations or steady-state concentrations), [A]_1_, [A]_2_, …, [A]_n_, will transform into a new set of values, [B]_1_, [B]_2_,…, [B]_n_, upon any perturbation, from altered enzyme activity to inhibitory drug effects. The percentage changes (PC) in the simulation output of the perturbed state relative to those of the initial state are of central interest.

(1) $$PC_{i}=\;100\;\times \frac{{\left[B\right]}_{i}-{\left[A\right]}_{i}}{{\left[A\right]}_{i}},\;i=\;1,2,\ldots ,n,$$

where PC_*i*_ in Equation (1) denotes the percentage change in the simulation value of the *i*th metabolite. CytoCopasi uses the PC values to customize the current network based on its deviation from the previously imported reference network.

The node size and color are changed based on deviations from the reference network. Node sizes are readjusted according to the absolute value of the percentage change for the corresponding metabolite. Metabolites, whose concentration profiles vary greatly upon perturbation, would therefore be represented with bigger nodes. In contrast, the metabolites with concentration profiles that did not vary across the two models become invisible in the final network. Metabolite upregulation and downregulation are depicted with the respective assignment of red or cyan colors to affected nodes. A feature where a cursor can be hovered over a node to bring percentage change, the initial, and the perturbed values into view, is available as well. This way of node resizing ensures that the metabolites affected by the applied changes are visually emphasized.

#### 2.3.1 Comparative simulation analysis between two models

**Figure 3.**
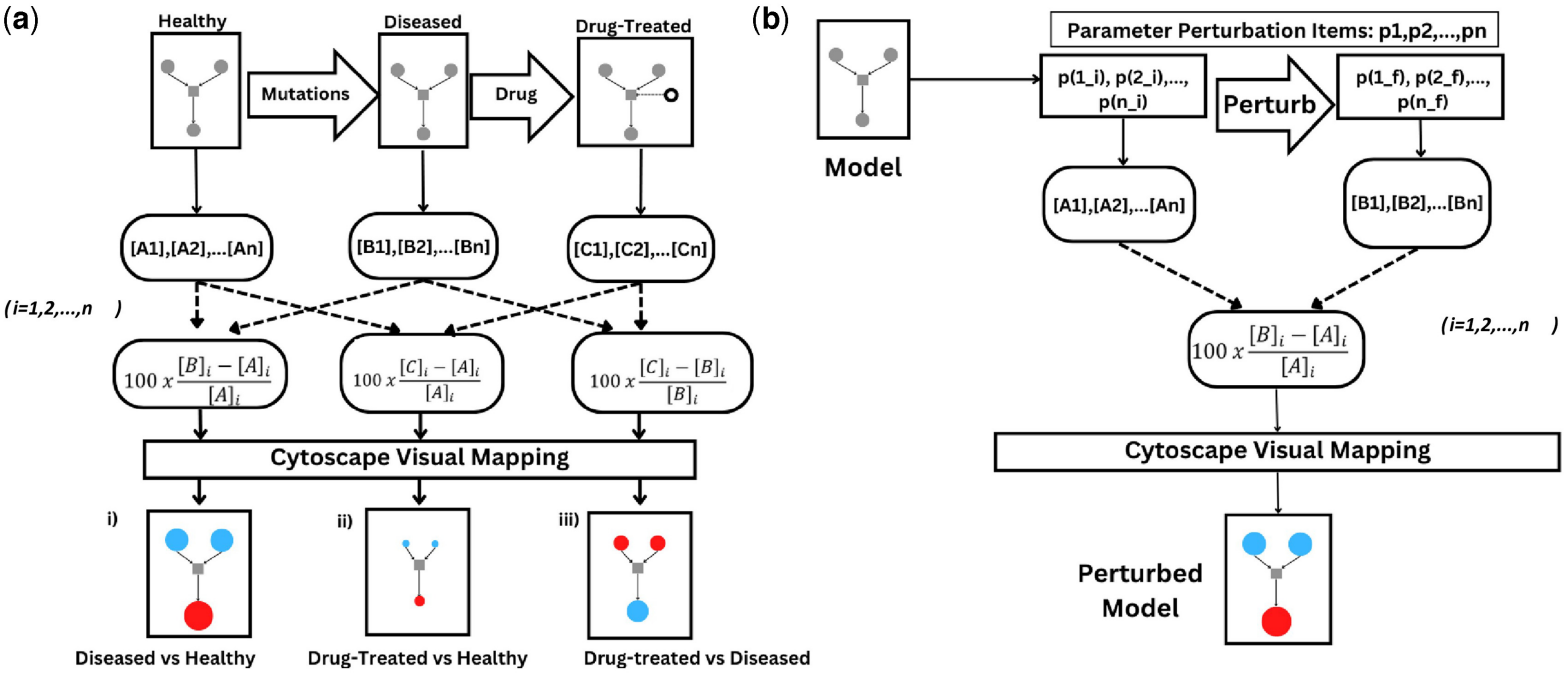
Illustration of (a) comparative simulation analysis that allows users to visualize the deviations in a COPASI model based on percentage changes in final concentrations relative to a reference COPASI model and (b) parameter perturbation that sequentially runs ODE simulations on the same model while tweaking parameters specified by the user. The algorithm computes the difference between the concentration profile of the final simulation with that of the initial simulation. In both panels (a) and (b), red signifies metabolite upregulation in the perturbed state, cyan denotes downregulation, and node size represents the magnitude of deviation.

The comparative modeling feature is illustrated in three scenarios (Fig. 3a) where simulations of percentage changes are computed between two separate models. The workflow initiates with importing a reference model and a perturbed model. Users can choose between a steady-state analysis or a time course simulation. Based on this choice simulations on each of the two models are simulated sequentially. The percentage changes of each metabolite are then automatically computed from the two sets of concentration data, displaying the resultant changes by node size and color on the second model.

In the first scenario, a comparative simulation between a healthy model and a disease model (Fig. 3a) is shown. Here, the disease model is an altered version of the healthy model where mutations were introduced by adding and/or removing reactions or changing enzyme parameters. The model comparison reveals the metabolite deviation in the diseased state relative to the healthy state (Fig. 3a, i). In the second scenario a comparison between the healthy model and the drug-treated disease model is made. If the drug successfully mitigates the differential metabolite concentrations of the diseased state, then the concentration profile of the drug treated model should resemble that of the healthy state, with the observation of only minor deviations (Fig. 3a, ii). In the third scenario, the disease model and its drug-treated version are compared. The latter model is generated by adding a drug metabolite and its corresponding reaction to the disease model. This comparison reveals the deviations from the disease state induced by drug treatment (Fig. 3a, iii).

#### 2.3.2 Parameter perturbations on the same model

Parameter perturbation allows the comparison between the original model and its perturbed state. Two ODE simulations are run sequentially, the first with the initial value of the scan item and the second with the perturbed value. Using COPASI’s parameter scan feature that supports multiparameter selection, it is possible to perturb multiple model values and observe the overall effect.

The initial and the perturbed values are stored and used to compute the percentage changes in the perturbed state relative to the initial state. (Fig. 3b).

## 3 Application

Using a reaction model comprising ERK, PI3K/Akt, and Wnt/β-catenin signaling networks and the crosstalk between them (Padala *et al.* 2017) CytoCopasi’s target discovery and drug evaluation capabilities are demonstrated. The activation dynamics of pERK, pAkt, and β-catenin/TCF, known hallmarks of tumor tissue proliferation because of further phosphorylation of transcription factors in the nucleus (Komiya and Habas 2008, Chakraborty *et al.* 2014), were investigated.

### 3.1 Comparative analysis of healthy and the cancerous signaling network

Mutations as described by Padala and co-authors (Padala *et al.* 2017) were introduced into the healthy signaling network to model the effect of aberrant signaling networks. In the Ras mutated model, reaction 8b responsible for deactivating Ras was removed. Similarly, BRaf mutation was generated by deleting reactions 17a and 17b involved in BRaf deactivation. The key difference between a healthy and aberrant signaling network is that phosphorylation of Raf1, ERK, and MEK is transient in the former and prolonged in the latter. This result reported by Padala *et al.* was discovered from individual time course simulations.

**Figure 4.**
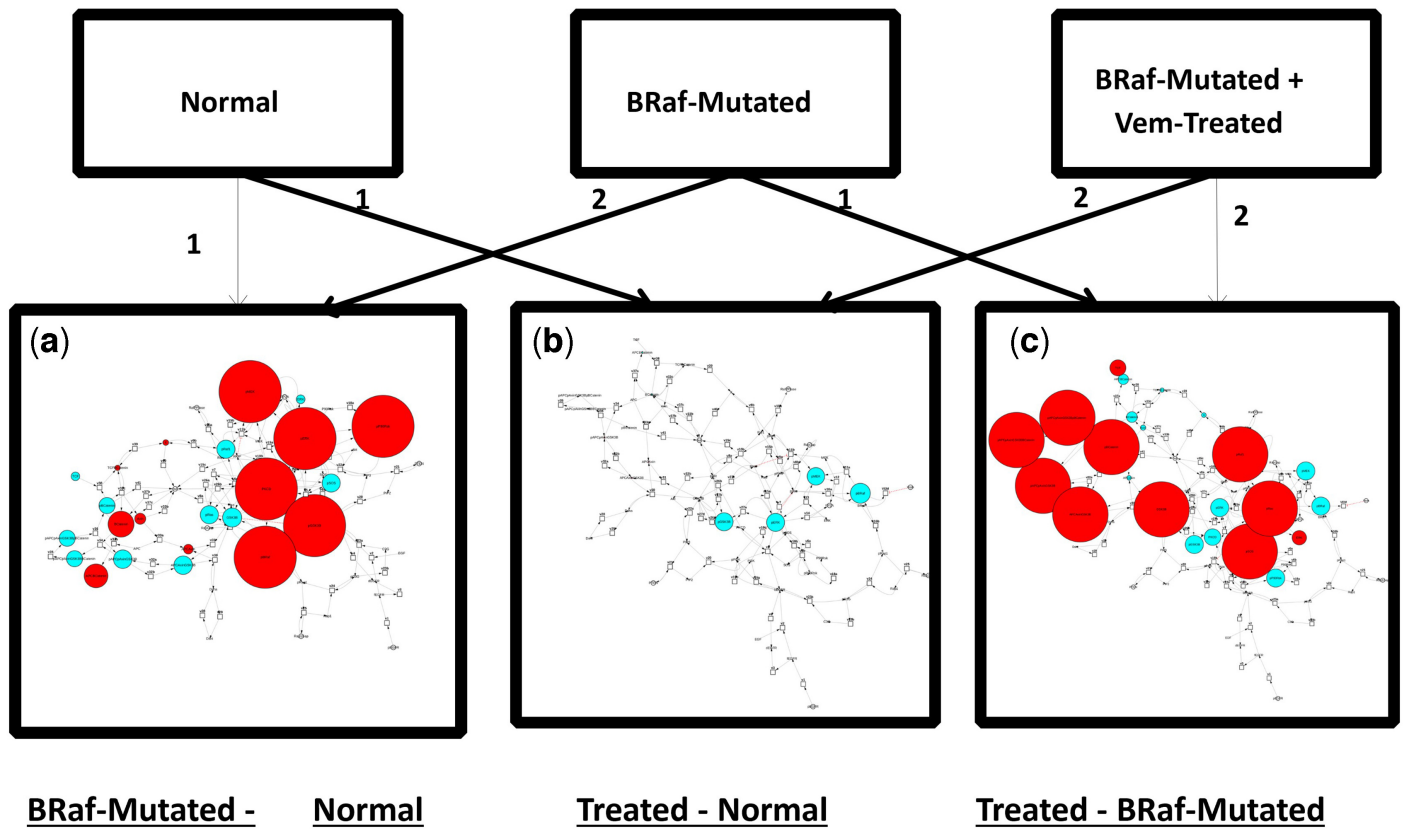
Application of comparative simulations displaying the variations between two networks. The numbers on arrows indicate the order of “Import + Run Simulation” actions. In each comparison, 1 indicates the reference model, while 2 indicates the model exhibiting deviations with respect to 1.

Using the CytoCopasi platform mutation-induced changes in metabolite concentrations are revealed through a comparative time-course simulation workflow. Here transient metabolite concentrations after 15 000 s were compared. Automatically, Cytoscape nodes representing metabolites affected by BRaf mutation were relatively resized by CytoCopasi to reflect changes in transient concentrations from the healthy signaling network (Fig. 4a). Phosphorylated forms of BRaf (pBRaf) and MEK (pMEK) exhibit a 10^6^% increase at the 15 000-s point in the simulation. The consecutive overactivation of pBRaf and pMEK lead to an increase in pERK activation by 5×10^5^%, which further induces GSK3β phosphorylation (pGSK3β) and paves the way for βCatenin accumulation. These results from a rapid visual analytics CytoCopasi approach agree with the observations from Padala *et al.*, including the positive feedback loop between the ERK and Wnt/β-catenin networks. This demonstrates that CytoCopasi has successfully reproduced the deterministic concentration profile of perturbed signaling networks through a seamless single platform process.

Further through the perturbation facility, the platform is able to monitor drug effectiveness. This is illustrated on two well-known BRaf inhibitors, Vemurafenib and Dabrafenib, that were independently introduced to the system. A reaction, where the drug compound facilitated the dephosphorylation of pBRaf to BRaf, deactivating the phosphorylated form was added to the system. The reaction mechanisms comprised a modified Michaelis Menten for Vemurafenib (Gianì *et al.* 2019) and reversible mass action for Dabrafenib (Hamis *et al.* 2021). The kinetic parameters were retrieved directly from the in-silico models. Detailed inhibitory kinetics for drug-treated models can be found in Supplementary Material S3, with kinetics constants and relevant literature summarized in Supplementary Table S1.

Two comparative calculations were run for each drug, and the results for Vemurafenib are shown in Fig. 4. The comparison was made between the drug-treated network and the healthy signaling network (Fig. 4b), with the premise that the drug should have brought the concentration profile of the cancerous network significantly close to that of the normal signaling network. The effect of drug intervention directly on the BRaf-mutated network was illustrated in Fig. 4c.

Treatment with Vemurafenib and Dabrafenib yielded similar results such that the comparison with the untreated disease model revealed a near-complete elimination in pBRaf. Subsequently, this caused a reduction of pMEK and pERK concentrations to 0.002% of their levels in the disease network. Further an approximate increase of 1500% in GSK3β was observed, exhibiting higher prevention of free βCatenin formation, which decreased by 60.3%. Upon superimposing the treated and healthy network models very few variations between the concentration profiles are observed. What is notable is that the phosphorylation of B-Raf, ERK, MEK, and GSK3β is significantly less in the drug-treated model than in the healthy model, while a moderate reduction in GSK3β is observed.

### 3.2 Direct visualization and analysis of the impact of model perturbations

**Figure 5.**
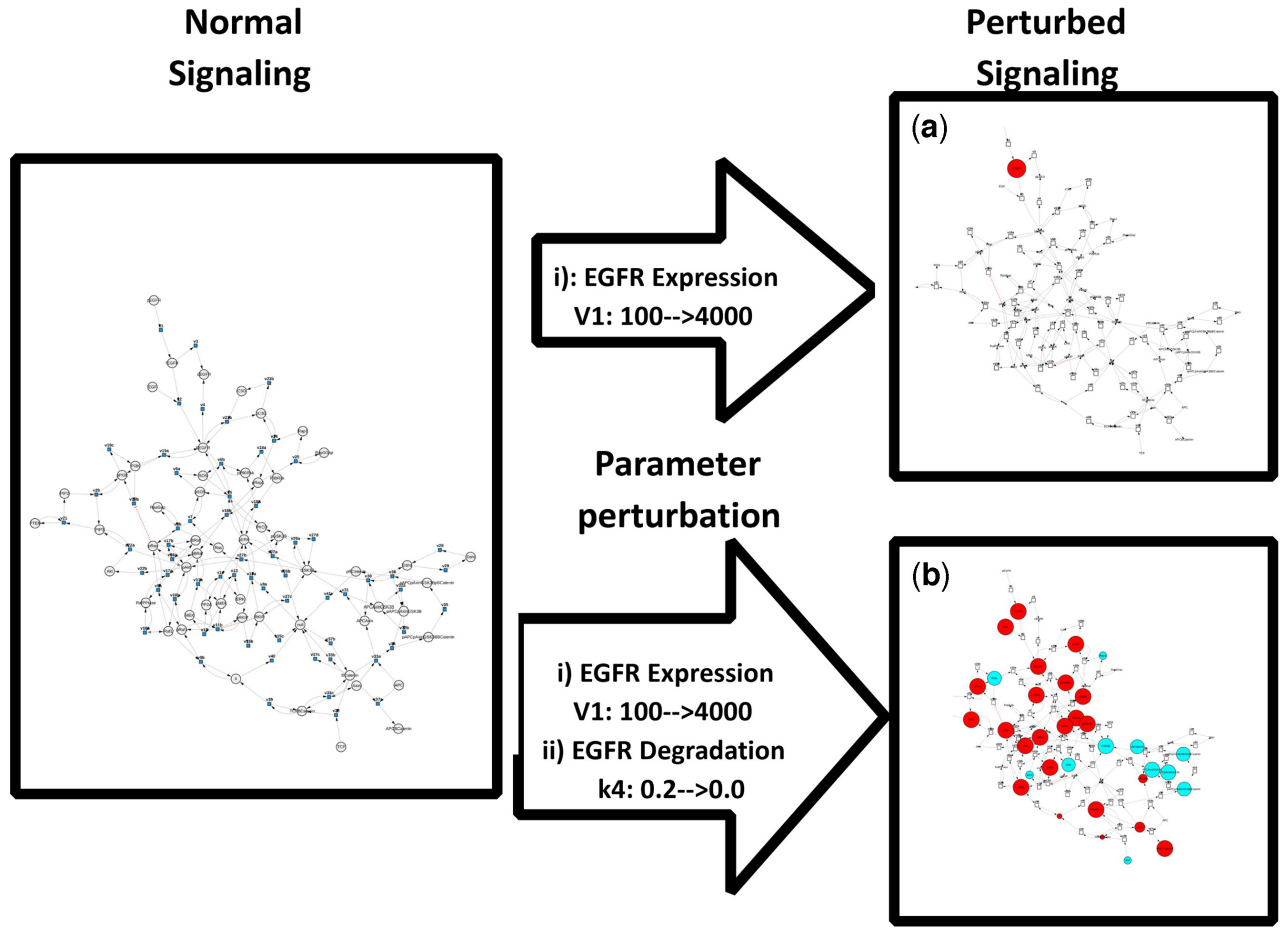
Illustrating parameter perturbations applied to normal models by varying EGFR expression. (a) Single parameter perturbation: increasing the expression of EGFR only shows no perturbation of the concentration profile. (b) Multiparameter perturbation with two parameters: increasing EGFR expression and reducing EGFR degradation showing aberrant signaling with increased concentrations in cancer hallmarks.

The effect of EGFR overexpression and mutation on the signaling network is undertaken making use of the novel deployment of the parameter perturbation functionality to simulations of altered experimental conditions. Previous studies using time course simulations concluded that overexpression of EGFR by 40-fold change did not alter pERK and pAkt concentrations due to the rapid degradation of EGF-bound EGFR. Here by running a single parameter perturbation in a 15 000 s time-course simulation for EGFR expression, we confirmed that EGFR expression alone does not alter the concentration profile (Fig. 5a).

Further in previous studies (Lynch *et al.* 2004, Sordella *et al.* 2004) it was shown, as well as computationally demonstrated in the original study (Padala *et al.* 2017), that the nullification of EGFR degradation led to a constitutive pERK and pAkt activation. Using CytoCopasi’s parameter perturbation functionality the complete mutation was simulated by simultaneously increasing EGFR expression 40-fold and decreasing the degradation reaction constant k_4_ from 0.2 to 0.0 nmol/s. This experiment revealed a significant increase in phosphorylated forms of BRaf, MEK, ERK, βCatenin, and Akt (Fig. 5b).

Consequently, expanding on the original study of EGFR mutation, we tested whether Vemurafenib would be effective in treating the signaling network having both BRaf and EGFR mutations. Starting with the untreated BRaf model with Vemurafenib added as a metabolite, but with an initial concentration of 0.0 uM, the network was scanned for three parameters:

- EGFR expression constant V_1_ increases from 100 to 4000.
- EGFR degradation constant k_4_ decreases from 0.2 to 0.0
- Vemurafenib's initial concentration increases from 0.0 to 1000 nM.

**Figure 6.**
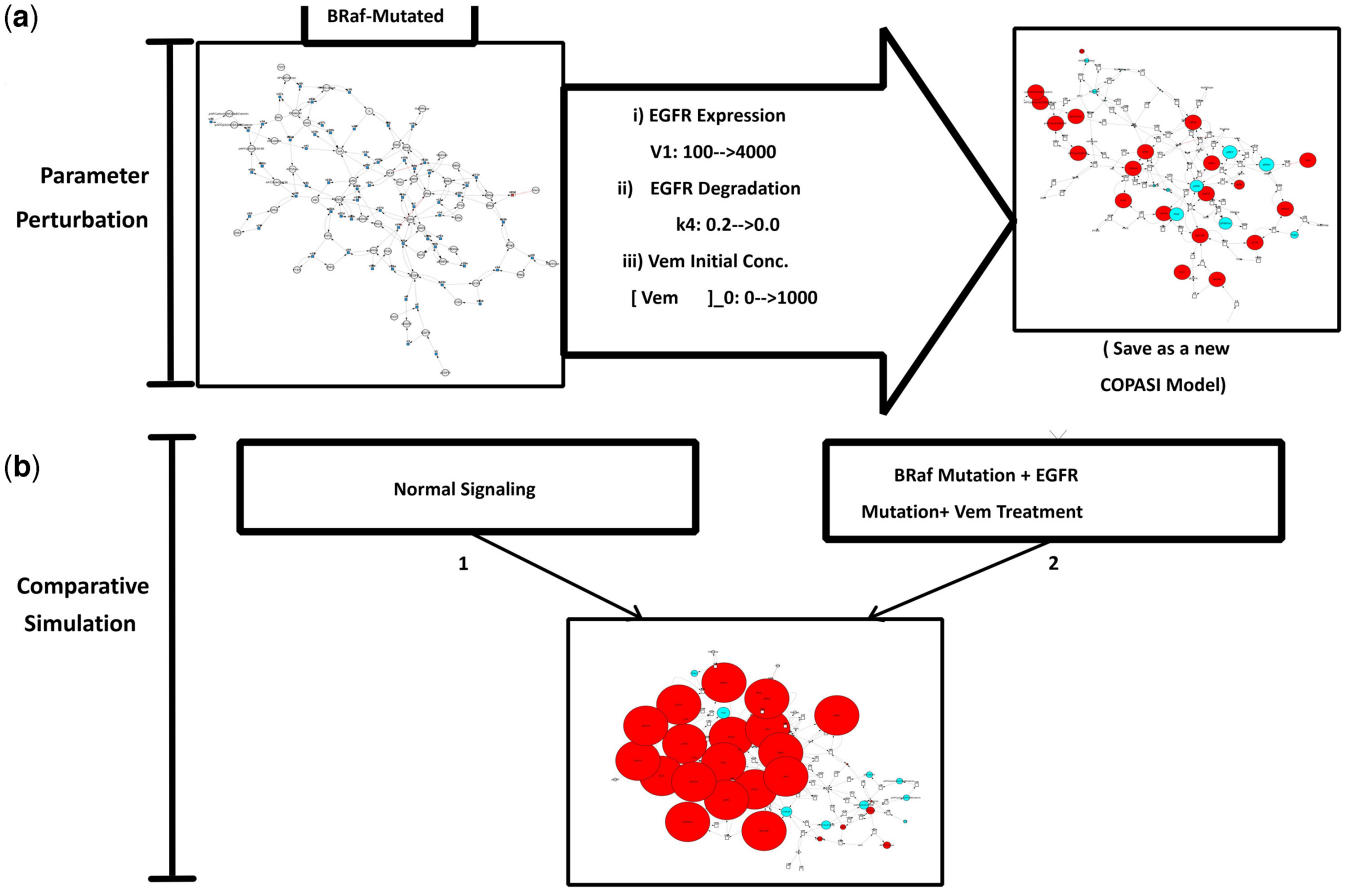
Illustration of a combination of Parameter Perturbation and Comparative Simulation. (**a**) A parameter perturbation on the BRaf Mutated model, (**b**) followed by the comparison of the perturbed model to the normal signaling model is shown to reveal that Vemurafenib is ineffective in the inhibition of pERK expression in the presence of EGFR mutations.

The model, using the three perturbed parameters, reveals that pAkt concentration undergoes the same increase even with Vemurafenib treatment (Fig. 6). This suggests that Vemurafenib may not be effective in altering pAkt overexpression. Furthermore, although the perturbations cause a reduction in pERK, the final concentration in this network is 23 nM, which is still 100-fold higher than the amount in the healthy signaling network. Running a comparative simulation between the perturbed network and the healthy network validates the findings of the multiparameter perturbation, where significant upregulation of the cancerous signaling network hallmarks is observed.

Overall, CytoCopasi leverages COPASI’s parameter scan functionality to unveil details of aberrant signaling upon simultaneous mutation of BRaf and EGFR, which is common to many cancer subtypes, including breast and ovarian cancer.

## 4 Discussion

CytoCopasi addresses two major drawbacks of current systems biology and specifically chemical systems biology implementations using ODE-based dynamic models. Firstly, the previously disconnected space between statistically based genome wide modeling, genome-scale metabolic modeling and dynamic biochemical reaction kinetics modeling is reduced. This is done by introducing CytoCopasi (a chemical systems biology computational package) as a novel Cytoscape (a genomic modeling platform) application in so enabling the future methods development of the transcriptomic translation of enzymatic action. Secondly a centralized platform for biochemical database-assisted high throughput model construction, parameterization, and large-scale visual analytics is absent in advanced systems drug testing and target discovery. To that end, CytoCopasi is introduced as a novel Cytoscape application that integrates model curation and simulation functionalities of standalone software and databases while harnessing the informative visualization mapping tools of Cytoscape’s core.

The utility of CytoCopasi was demonstrated through a set of systems biology studies of varying complexities that confirmed the successful incorporation of COPASI in CytoCopasi. This was made possible by using the platform’s direct access to pathway and enzyme parameter databases KEGG and BRENDA in accelerated and error-minimized model curation illustrations. The integration of these two databases can significantly reduce the time taken to construct representations of biological systems lacking previous computational modeling efforts.

Leveraging Cytoscape’s capability to open multiple networks in one session, systems biology workflows were constructed to visualize concentration profile shifts in signal transduction upon perturbations, and the results agreed with the original systems biology study. In addition, parameter perturbation and comparative simulation were combined to generate novel insight. Specifically, it was first demonstrated through multiparameter perturbation that a chemotherapeutic agent could still alleviate aberrantly activated signal transduction with multiple mutations. Nevertheless, a subsequent comparison the healthy network revealed that the inhibition was not effective enough to bring the signaling hallmarks back to their normal values. Thus, the inadequacy of single-point inhibition in systems harboring multiple mutations was unveiled. Following the comparative approach, the effects of multiple therapeutics can be monitored computationally, paving the way for the development of more advanced treatments for complex diseases.

The integration of COPASI into Cytoscape results in an expansive visualization platform able to present a holistic representation of perturbation effects is provided. Tedious scripting and spreadsheet manipulation needed to visualize the results have been made obsolete through a single multiuse platform. Simultaneous displays of variations for every metabolite collectively done in the same Cytoscape session make large-scale data analytics accessible. Through this dynamic kinetics modeling Cytoscape application novel regulatory experiments can be routinely performed removing the need for multiple targeted time course simulations.

Computer-aided target and drug discovery is of central importance to personalized medicine research. Developing therapeutics with higher potency, minimum required dose, long-term treatment success, and minimum off-target effects requires rapid biochemical network response simulations. This systems biology, specifically ODE-based dynamic analysis of biochemical reaction networks, benefits from uninterrupted communication between Cytoscape visual tools, large databases, and COPASI’s simulation capabilities. Central to large scale studies needed to build complex biological network models is the reproducibility and sharing of developed workflows and data acquisition between laboratories made possible by the CytoCopasi platform.

## Supplementary Material

btad745_Supplementary_Data

## Data Availability

The COPASI files used for illustrative purposes in Sections 2.2.3, 3.1, and 3.2 are available at (https://github.com/scientificomputing/CytoCopasi). The original SBML model from Lambeth and Kushmerick (2002) and relevant kinetic parameters can be downloaded from (https://www.ebi.ac.uk/biomodels/MODEL6623617994). The SBML models from Padala *et al.* (2017) are available at (https://www.ebi.ac.uk/biomodels/BIOMD0000000655).
