# Supplementary material for: CytoCopasi: a chemical systems biology target and drug discovery visual data analytics platform
Source: Bioinformatics. 2023 Dec 9;39(12):btad745. doi: 10.1093/bioinformatics/btad745 (PMC10963058; doi:10.1093/bioinformatics/btad745)
Supplement: btad745_Supplementary_Data [file btad745_supplementary_data.docx]

CytoCopasi: A Chemical Systems Biology Target and Drug Discovery Visual Data Analytics Platform

Emre Kaya^1,2^ and Kevin J. Naidoo^1,2,*^

^1^Scientific Computing Research Unit Address, PD Hahn Building, University of Cape Town, Rondebosch 7701,

^2^Department of Chemistry, PD Hahn Building, University of Cape Town, Rondebosch 7701.

___________________________________________________________________________________________________

# 1- Step-by-step Model Construction for Fuentes et al. [1] (BIOMD0000000092)

The steps for model construction have been described in detail in User Manual sections 4.1- 4.4

- Click on “New Copasi Model”. A new window will appear. Give a name to your model and select the following:

Time Unit: s; Volume Unit: l ; Quantity Unit: mol (these units will apply to all the metabolites and reactions to be added)

Warning: When you add parameters in reactions, enter the values WITHOUT the units.

**Reaction r1**

- An empty Cytoscape network has been created and new buttons appear at the bottom of the CytoCopasi panel: Click on “Add Reaction”

z=e+w

(click on Syntax Guideline for tips on the correct formatting of chemical equations)

- Click Commit (a warning appears to indicate that a metabolites of the reaction are assigned to a default compartment – because we did not create a custom one).
  - The pop-up about compartment creation may appear in the background (for Mac users).
- The rate law will be defined by the user. Click on “New rate law” to open the pop-up for custom rate law creation. Enter the following

Function Name: Function for r1

Formula: k1*z ;

- Click commit and assign the roles of the variables. “k1” is a parameter and “z” is the substrate in r1.
- Click add. The new function has now been defined.
- Next, click parameters and enter the following for k1

k1 = 0.004 l/s

- Click Add.

| 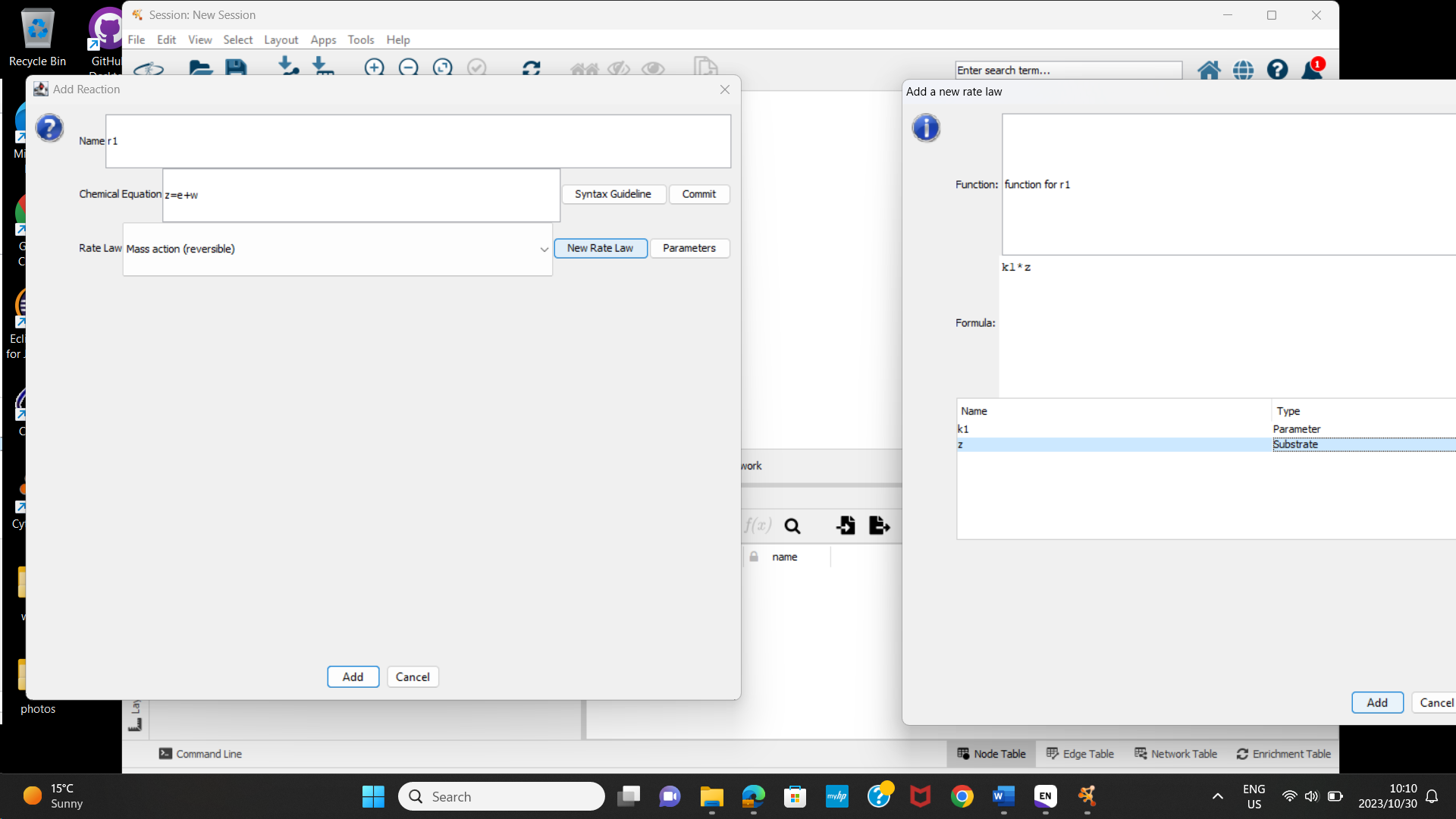 | 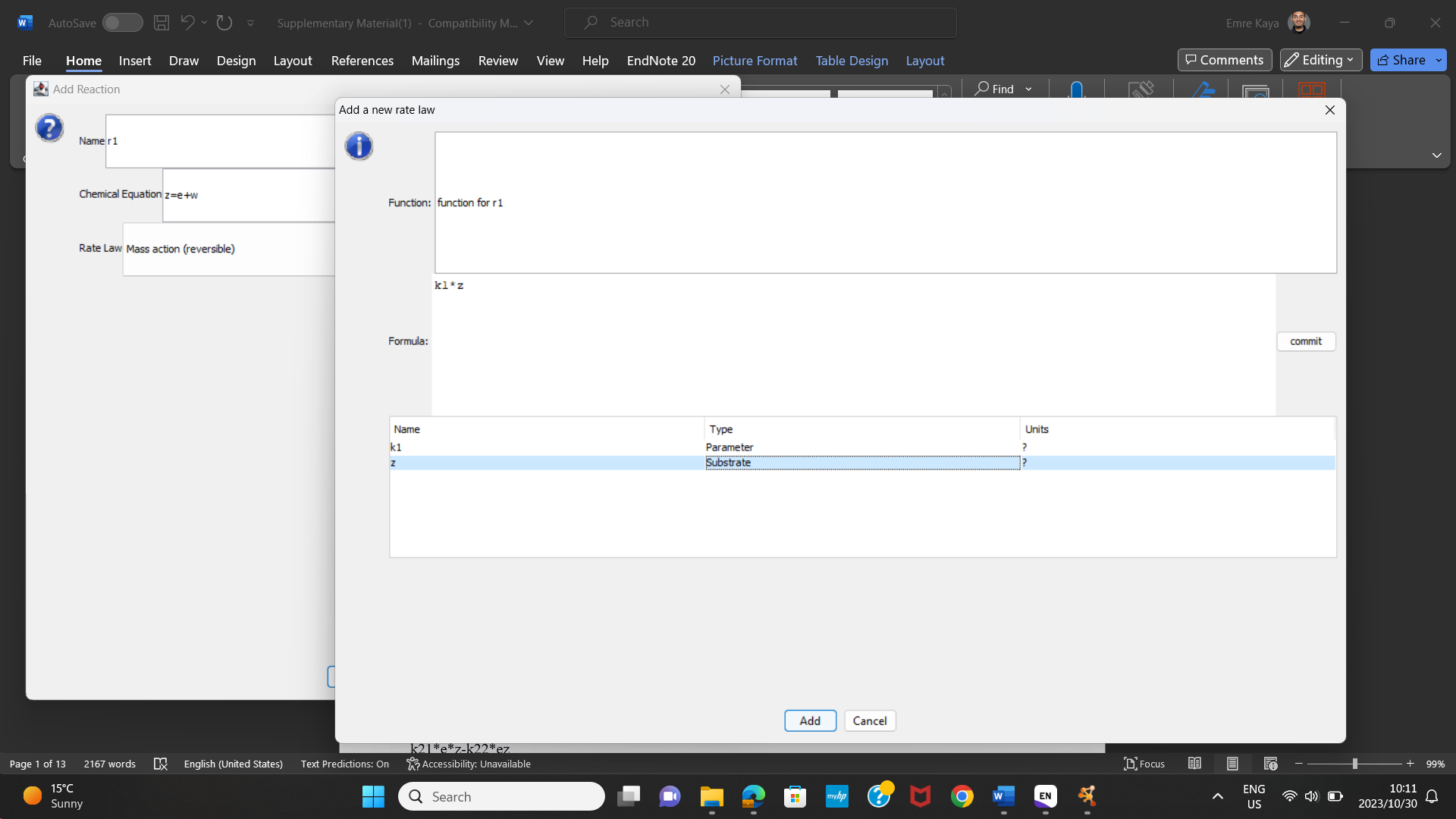 |
| --- | --- |
| 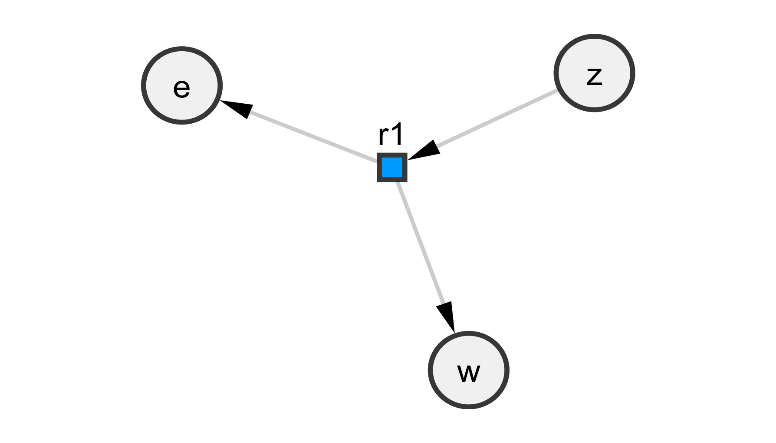 | |
| **Figure S1**: The reaction specifics for r1 entered into CytoCopasi and the resulting network view | |

**Reaction: r2**

e+z=ez

Function for r2: mass action (reversible) (No need to create a custom rate law, this is already available in COPASI’s function database.

- Find Mass Action (reversible) and then click Parameters

k1 = 1000 l^2^/(mol*s)

k2 = 0.00021 l/s

**Reaction: r3**

ez = 2*e+w

Function for r3:

k3*ez; k3 = 0.00054 l/s

| 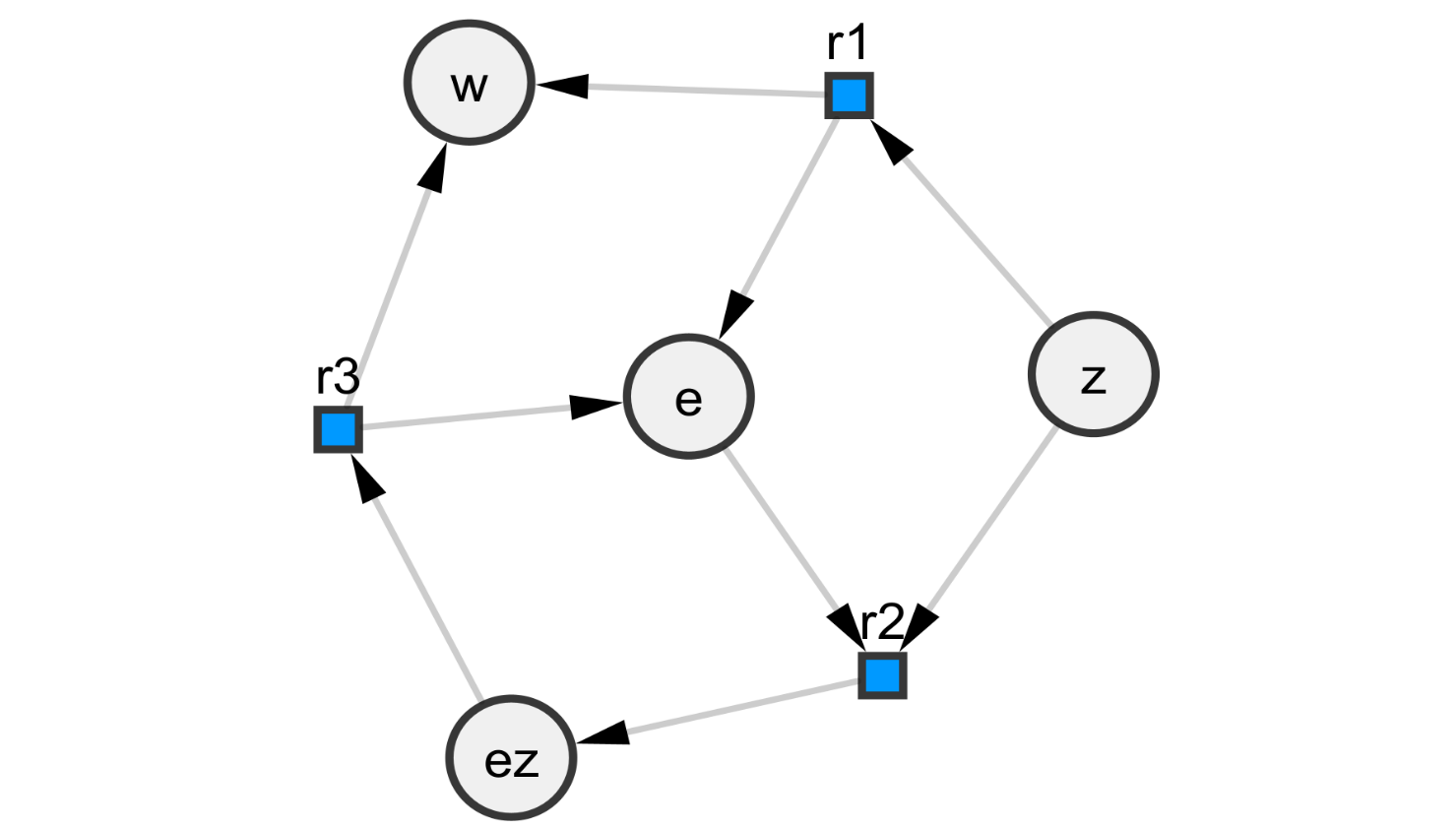 |
| --- |
| **Figure S2:** The reaction specifics for r3 entered into CytoCopasi and the resulting network view including all three reactions |

When reactions are created, initial metabolite concentrations are set to 1.0 and status is set to “Reactions” by default. Double-click on the metabolite you want to edit. Change the initial concentrations as follows:

[e]_0_, [ez]_0_, and [w]_0_ = 0.0 mol/l

[z]_0_ = 2.4e-5 mol/l

Now, run the time course with 100 seconds and 100 intervals to confirm that you constructed the model correctly.

- Click Time Course Simulation on the main panel.
- Enter the following:
  - Duration: 100
  - Intervals: 100
  - Start Output Time: 0.0
- Select Output Assistant to select the metabolites you want to monitor.
- To select multiple metabolites, hold CTRL and select the metabolites.


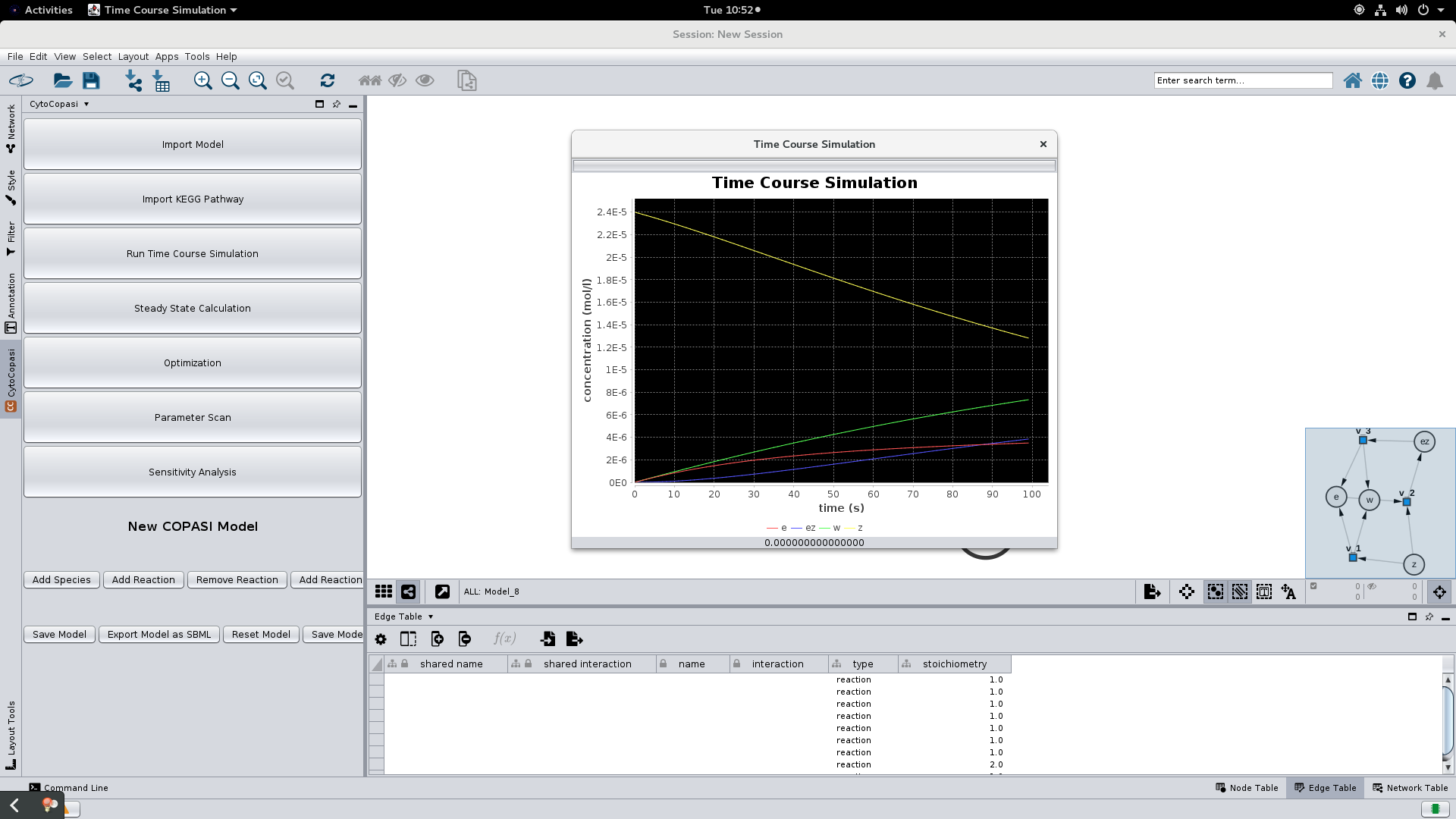


**Figure S3**: Running a time course simulation for 100 seconds to display the concentration profiles of the metabolites (note: the background has been changed to black manually for ease of visualizing the curves

## 2- Replicating Lambeth et al. [2] from the KEGG Glycolysis Model

The steps for editing a reaction network fit into one of the four categories below:

1. **Removing a reaction**: Click on “Remove Reaction” from CytoCopasi’s panel, and select the reaction you want to remove.
2. **Adding a reaction to the imported model** – Click on “Add Reaction” in the CytoCopasi panel and enter the specifics (name, formula, rate law, and parameters). This will create a new reaction node.
3. **Change the chemical equation of an existing reaction node:** Double click on the reaction node and select “edit reaction” which creates a pop-up for changing the chemical equation of the reaction. (e.g., for changing a=b to a+x=b+y)
4. **Modifying the rate law or parameters:** Double click on the reaction node and select “edit rate law” to change the rate law, define a custom rate law, or change the parameter values for an existing rate law.
   1. **Note:** if you define a custom rate law, an Apply button will appear on the rate law panel. Click on this button before clicking on “Ok”.

The original SBML file


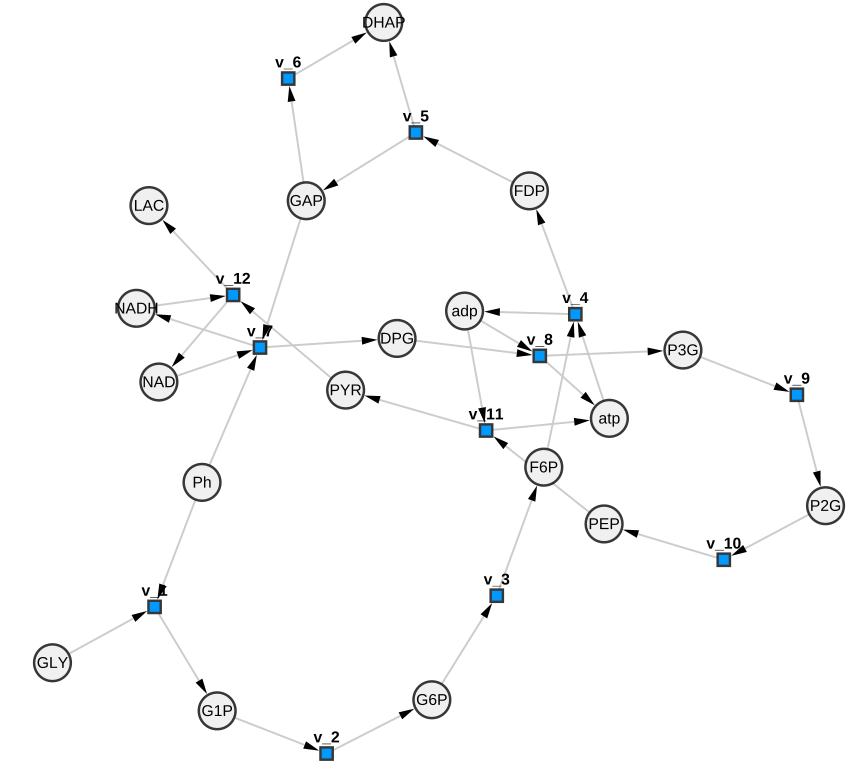


**Figure S4**: The Cytoscape network of the original model

- Click Import KEGG Pathway and find Glycolysis/Glucogenesis. Click Load
  - If the imported network is empty or incomplete, this is likely a timeout issue. In that case, you should try loading the pathway again.

**Generate Subnetwork:**

Click on Generate Subnetwork the button at the bottom of the CytoCopasi Panel and Select “Glycolysis (Embden-Meyerhof pathway)”


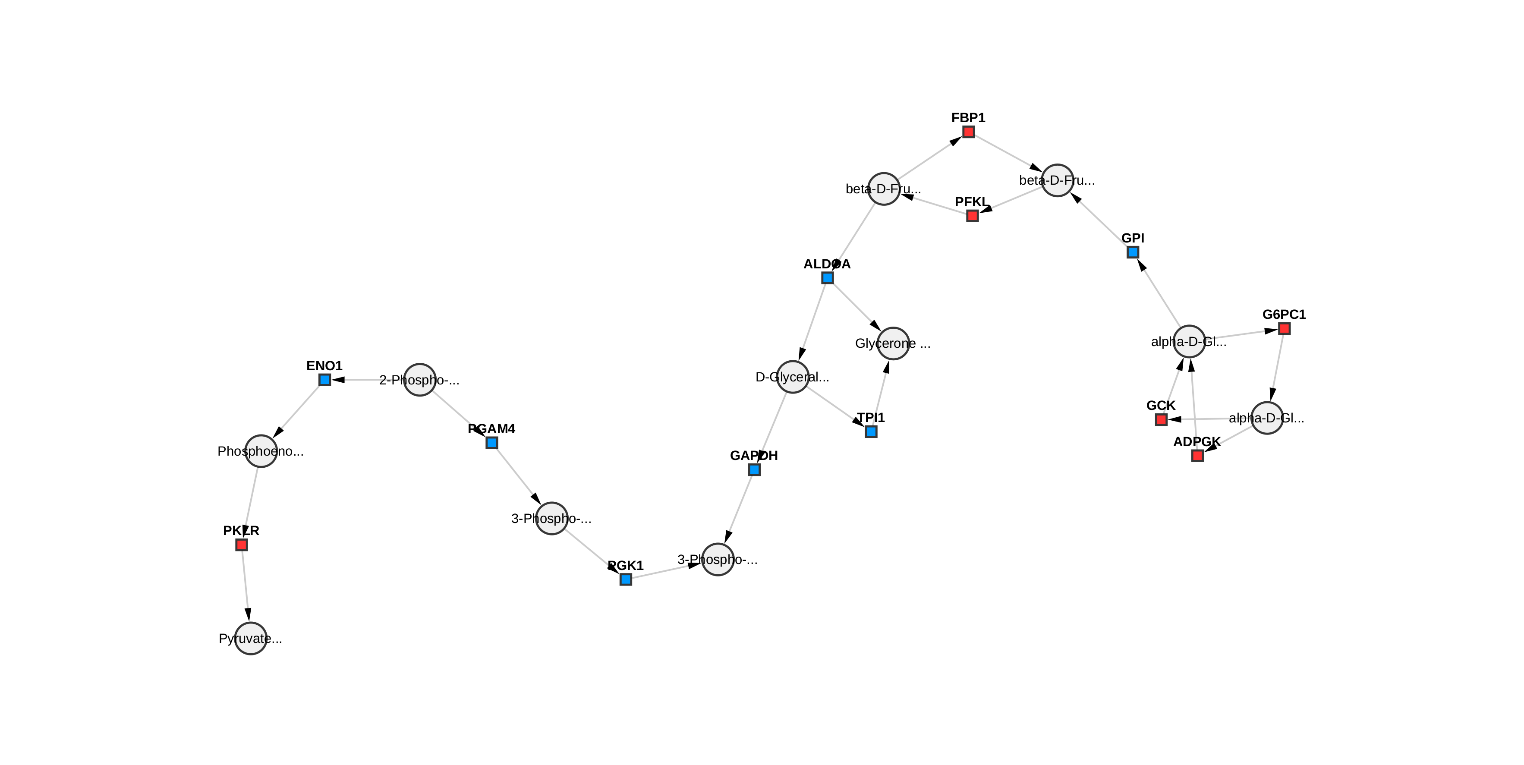


**Figure S5**: The KEGG Glycolysis module M00001 Glycolysis (Embden-Meyerhof pathway)

### 2.1 Metabolite Names

**Step 1: Rename the Metabolites:** Double click on each metabolite and change the names

- This step is optional, but done here to create an exact replicate of the original SBML model. If you want to continue without changing the names, use KEGG IDs to change chemical equations or add reactions. The KEGG ID for a KEGG metabolite can be found under the “shared name” column in the node table.

WARNING: Changes to node attributes (e.g., metabolite names, initial concentrations, chemical equations, etc) should always be made through CytoCopasi’s visual interface (i.e., by double-clicking on the nodes to open Node Editing Dialogs). Refrain from modifying attributes on the Cytoscape Node Table.

Nomenclature:

GLY: glycogen

G1P: alpha-D-Glucose

G6P: Alpha-D-Glucose 6-phosphate

F6P: D-fructose 6-phosphate

FBP: D-fructose 1,6-bisphosphate

DHAP: glycerone phosphate (1,3-dihydroxyacetone phosphate)

GAP: D-glyceraldehyde 3-phosphate

DPG: 3-phospho-D-glyceryl phosphate

P3G: 3-phospho-D-glycerate

P2G: 2-phospho-D-glycerate

PEP: Phosphoenolpyruvate

PYR: Pyruvate

LAC: Lactate

P_h_: inorganic phosphate

| 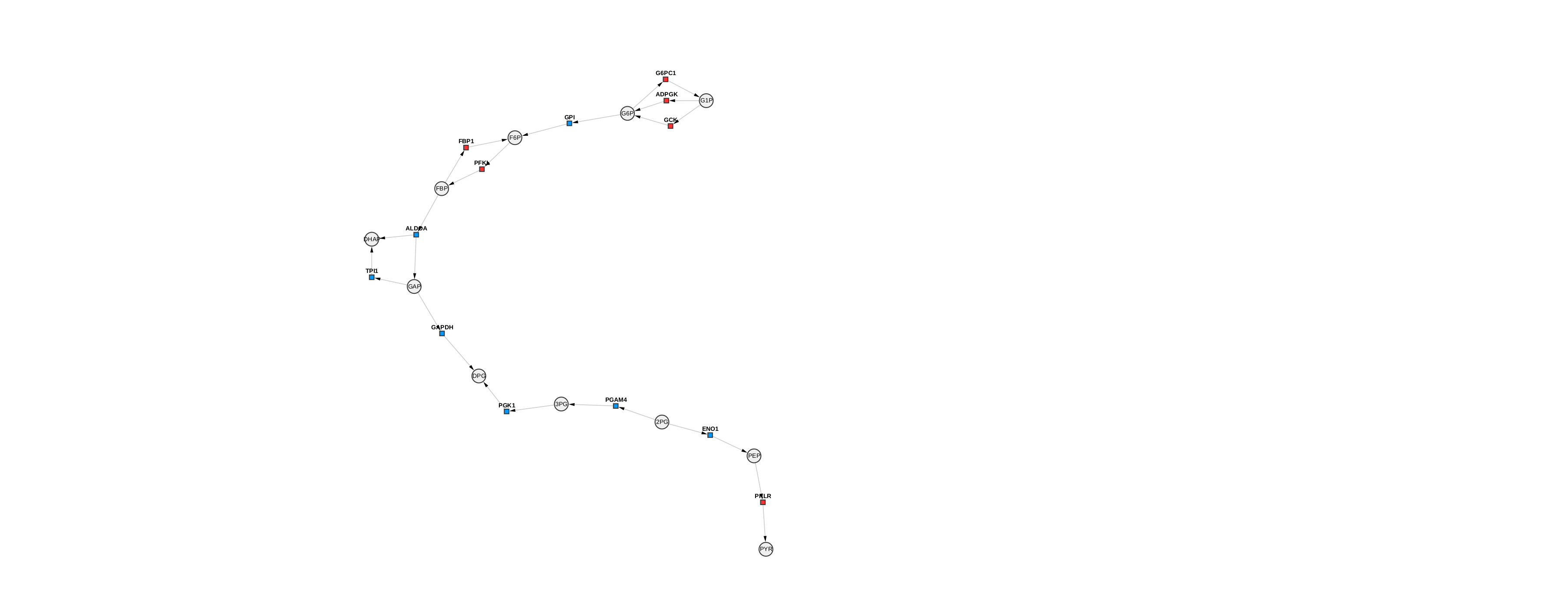 |
| --- |
| **Figure S6**: Metabolites have been renamed for ease of visualizing and customization |

### 2.2 ADDING/REMOVING REACTIONS, CHANGING REACTION CHEMICAL EQUATIONS TO ADD NEW METABOLITES

Notes:

- All the kinetics parameters are given in mM unless stated otherwise
- You can copy-paste the rate laws from this document into the rate law text area. If you get a “check your syntax error” after clicking commit, make sure that there are no spaces at the end of the rate law formula.
- Detailed information about looking up parameter values via BRENDA can be found in CYTOCOPASI APP MANUAL – Section 4.4.4
- parameters missing from BRENDA– mainly Vmax values – were completed using the original study.

**Step 2:** **Remove the reactions between G6P and** **G1P**

**Step 3: Add v_2**

Chemical Equation: G6P=G1P

Rate Law: ((Vfpglm*G1P/Kpglmg1p)-(Vfpglm*Kpglmg6p/(Kpglmg1p*16.62))*G6P/Kpglmg6p)/(1 + G1P/Kpglmg1p + G6P/Kpglmg6p)

Brenda Keyword: Phosphoglucomutase

EC to select: 5.4.2.2

It did not return parameters for Oryctolagus cuniculus (rabbit) nor Homo sapiens (human), therefore we pulled both parameters from the paper.

Kpglmg1p= 0.063 [3]

Kpglmg6p= 0.03 [3]

**Step 4: Add v_1**

Ph+GLY=G1P

fracA*((Vfgly*Ph*GLY/(KgpAigly*KgpApi))/(1 + GLY/KgpAglyf + Ph/KgpApi + GLY*Ph/(KgpAglyf*KgpAipi) + GLY/KgpAglyb + G1P/KgpAg1p + GLY*G1P/(KgpAig1p*KgpAglyb)) - ((Vfgly*KgpAglyb*KgpAig1p/(KgpAigly*KgpApi*0.31))*G1P*GLY/(KgpAglyb*KgpAig1p))/ (1 + GLY/KgpAglyf + Ph/KgpApi + GLY*Ph/ (KgpAglyf*KgpAipi) + GLY/KgpAglyb + G1P/KgpAg1p + GLY*G1P/(KgpAig1p*KgpAglyb))) + fracB*((((amp^nH)/((Kgpamp^nH)*0.02))/ (1 + (amp^nH)/((Kgpamp^nH)*0.02))*(Vfgly*Ph*GLY/(KgpBiglyf*KgpBpi))/((1 + GLY/KgpBipi + Ph/KgpBiglyf + GLY/KgpBiglyb + G1P/KgpBig1p + GLY*Ph/(KgpBiglyf*KgpBpi) + GLY*G1P/(KgpBg1p*KgpBiglyb)))) - (((amp^nH)/((Kgpamp^nH)*0.02))/(1 + (amp^nH)/((Kgpamp^nH)*0.02))*((Vfgly*KgpBg1p*KgpBiglyb/(KgpBiglyf*KgpBpi*0.31))*G1P*GLY/(KgpBg1p*KgpBiglyb))/((1 + GLY/KgpBipi + Ph/KgpBiglyf + GLY/KgpBiglyb + G1P/KgpBig1p + GLY*Ph/(KgpBiglyf*KgpBpi) + GLY*G1P/(KgpBg1p*KgpBiglyb)))))

Keyword: Glycogen Phosphorylase

EC to select: 2.4.1.1

The K_m_ values are pulled entirely from BRENDA, while the K_i_ values for Glycogen Phosphorylase B are from BRENDA, and those for Glycogen Phosphorylase A are from the paper.

K_m_:

KgpApi: 28 [4]

KgpAglyf: 0.62 [5]

KgpAglyb: 0.15 [5]

KgpAg1p: 1.7  [6]

Kgpamp: 0.074 [7]

KgpBpi: 11 [7]

KgpBg1p: 5.5 [7]

K_i_: look at the commentary column to see what the inhibitor is being used against

KgpBipi= 0.71 [7]

KgpBig1p = 0.44 [7]

KgpBiglyf = 0.0028 [8]

KgpBiglyb = 5.9  [9]

We did not find inhibition constants for Glycogen Phosphorylase A, but the papers that contain Km constants can be checked by the user to see if they contain inhibition constants as well. For now, we are going to use the values from the original paper.

KgpAigly = 2 [10]

KgpAiPi = 4 [10]

KgpAig1p = 10.1 [10]

**Step 5: The reaction F6P=FBP is defined as one reversible reaction in the SBML model, whereas Kegg has a separate reaction node for the reverse reaction (because of different enzymes being involved).**

**So:** **Delete FBP1 and change the chemical equation for PFKL**

F6P+atp=FBP+adp

(Vfpfk*atp*F6P*(1 + (Kpfkatp*Kpfkf6p*Lo*(1 + (en*amp)/Kpfkamp)^4*(1 + atp/Kpfkiatp)^4*((1 + atp/KpfkatpT)*(1 + F6P/Kpfkf6pT) + FBP/KpfkfdpT + (adp*(1 + FBP/KpfkfdpT))/KpfkadpT)^3)/(KpfkatpT*Kpfkf6pT*(1 + amp/Kpfkamp)^4*(1 + (dn*atp)/Kpfkiatp)^4*((1 + atp/Kpfkatp)*(1 + F6P/Kpfkf6p) + FBP/Kpfkfdp + (adp*(1 + FBP/Kpfkfdp))/Kpfkadp)^3)))/(Kpfkatp*Kpfkf6p*((1 + atp/Kpfkatp)*(1 + F6P/Kpfkf6p) + FBP/Kpfkfdp + (adp*(1 + FBP/Kpfkfdp))/Kpfkadp)*(1 + (Lo*(1 + (en*amp)/Kpfkamp)^4*(1 + atp/Kpfkiatp)^4*((1 + atp/KpfkatpT)*(1 + F6P/Kpfkf6pT) + FBP/KpfkfdpT + (adp*(1 + FBP/KpfkfdpT))/KpfkadpT)^4)/((1 + amp/Kpfkamp)^4*(1 + (dn*atp)/Kpfkiatp)^4*((1 + atp/Kpfkatp)*(1 + F6P/Kpfkf6p) + FBP/Kpfkfdp + (adp*(1 + FBP/Kpfkfdp))/Kpfkadp)^4))) - (0.004117429077284144*Vfpfk*adp*FBP*(1 + (Kpfkatp*Kpfkf6p*Lo*(1 + (en*amp)/Kpfkamp)^4*(1 + atp/Kpfkiatp)^4*((1 + atp/KpfkatpT)*(1 + F6P/Kpfkf6pT) + FBP/KpfkfdpT + (adp*(1 + FBP/KpfkfdpT))/KpfkadpT)^3)/(KpfkatpT*Kpfkf6pT*(1 + amp/Kpfkamp)^4*(1 + (dn*atp)/Kpfkiatp)^4*((1 + atp/Kpfkatp)*(1 + F6P/Kpfkf6p) + FBP/Kpfkfdp + (adp*(1 + FBP/Kpfkfdp))/Kpfkadp)^3)))/(Kpfkatp*Kpfkf6p*((1 + atp/Kpfkatp)*(1 + F6P/Kpfkf6p) + FBP/Kpfkfdp + (adp*(1 + FBP/Kpfkfdp))/Kpfkadp)*(1 + (Lo*(1 + (en*amp)/Kpfkamp)^4*(1 + atp/Kpfkiatp)^4* ((1 + atp/KpfkatpT)*(1 + F6P/Kpfkf6pT) + FBP/KpfkfdpT + (adp*(1 + FBP/KpfkfdpT))/KpfkadpT)^4)/((1 + amp/Kpfkamp)^4*(1 + (dn*atp)/Kpfkiatp)^4*((1 + atp/Kpfkatp)*(1 + F6P/Kpfkf6p) + FBP/Kpfkfdp + (adp*(1 + FBP/Kpfkfdp))/Kpfkadp)^4)))

Keyword: Phosphofructokinase

EC to select: 2.7.1.11

It is best to pull the values from the original paper since all the values were obtained from rabbit.

Kpfkf6p: 0.18 [11]

Kpfkf6pT= 20 [11]

Kpfkatp= 0.08 [11]

KpfkatpT= 0.25 [11]

Kpfkiatp= 0.87 [11]

KpfkFDP= 4.02 [12]

KpfkFDPT= 4.02 [12]

Kpfkadp= 2.7 [12]

KpfkadpT= 2.7 [12]

Kpfkamp = 0.06 [12]

**Step 6:** **Change Reaction for GAPDH (GAP=DPG**)

GAP+NAD+Ph=DPG+NADH

((-11.235955056179776*Vfgad*DPG*NADH)/(Kgapdhgap*Kgapdhnad*Kgapdhpi) + (Vfgad*GAP*NAD*Ph)/(Kgapdhgap*Kgapdhnad*Kgapdhpi))/(1 + DPG/Kgapdh13dpg + GAP/Kgapdhgap + NAD/Kgapdhnad + (GAP*NAD)/(Kgapdhgap*Kgapdhnad) + NADH/Kgapdhnadh + (DPG*NADH)/(Kgapdh13dpg*Kgapdhnadh) + Ph/Kgapdhpi + (GAP*NAD*Ph)/(Kgapdhgap*Kgapdhnad*Kgapdhpi))

Keyword: Glyceraldehyde-3-Phosphate Dehydrogenase

EC to select: 1.2.1.12

Rabbit: **80% complete with BRENDA, one value from the original paper**

Kgapdhnadh=0.012 [13]

Kgapdhnad = 0.06 [13]

Kgapdhgap = 0.82 [13]

Kgapdh13dpg = 0.13 [13]

From paper:

Kgapdhpi = 0.29 [14]

**Step 7:** **Change Reaction PGK1 (DPG=P3G)**

DPG+adp=P3G+atp

((57109*Vbpgk*adp*DPG)/(Kpgk3pg*Kpgkatp) - (Vbpgk*atp*P3G)/(Kpgk3pg*Kpgkatp))/(1 + adp/Kpgkadp + atp/Kpgkatp + DPG/Kpgk13dpg + (adp*DPG)/(Kpgk13dpg*Kpgkadp) + P3G/Kpgk3pg + (atp*P3G)/(Kpgk3pg*Kpgkatp))

Keyword: Phosphoglycerate Kinase

EC to select: 2.7.2.3

All the values are pulled from BRENDA (for Homo sapiens)

Kpgk3pg = 0.1 [15]

Kpgkatp = 0.11 [15]

Kpgkadp = 0.1 [15]

Kpgk13dpg = 0.0077 [15]

**Step 8:** **Change Reaction PKLR (PEP->PYR)**

PEP+adp=PYR+atp

(Vfpk*PEP*adp/(Kpkpep*Kpkadp)-(Vfpk*Kpkpyr*Kpkatp/(Kpkpep*Kpkadp*10304))*PYR*atp/(Kpkpyr*Kpkatp))/(1+PEP/Kpkpep+adp/Kpkadp + PEP*adp/(Kpkpep*Kpkadp) + atp/Kpkatp + PYR/Kpkpyr + PYR*atp/(Kpkpyr*Kpkatp))

Keyword: Pyruvate Kinase, organism: Oryctolagus cuniculus

EC no to select: 2.7.1.40

The K_m_ values for PEP and ADP are pulled from BRENDA, and those for PYR and ATP are from the original paper.

Kpkpep = 0.076 [16]

Kpkadp = 0.357 [16]

Kpkpyr = 7.05 [17]

Kpkatp = 1.13 [17]

**Step 9:** **Add LACDH, the Reaction for Lactate**

PYR+NADH=LAC+NAD

((Vfldh*PYR*NADH/(Kldhpyr*Kldhnadh))-((Vfldh*Kldhlac*Kldhnad/(Kldhpyr*Kldhnadh*16198))*LAC*NAD/(Kldhlac*Kldhnad)))/(1 + PYR/Kldhpyr + NADH/Kldhnadh + PYR*NADH/(Kldhpyr*Kldhnadh) + LAC/Kldhlac + NAD/Kldhnad + LAC*NAD/(Kldhlac*Kldhnad))

Keyword: Lactate dehydrogenase; Organism: Homo sapiens

EC no: 1.1.1.28

3 out of 4 parameters were available on BRENDA:

Kldhpyr= 0.398 [18]

Kldhnad = 0.5 [19]

Kldhlac = 10.73 [19]

From the original paper:

Kldhnadh = 0.002 [20]

**Final view (with Hierarchical Layout instead of force-directed)**


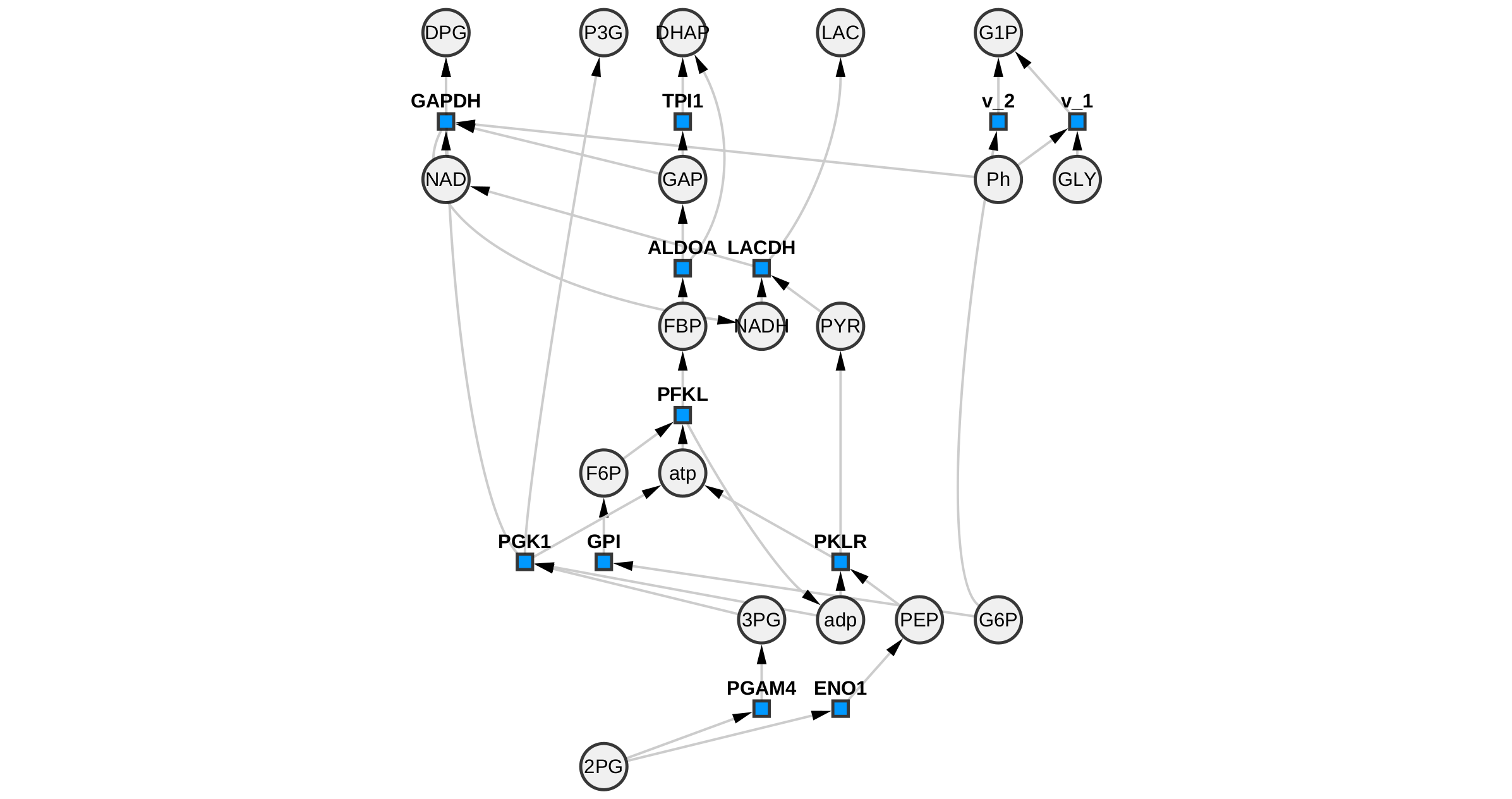


**Figure S7**: Final view (with Hierarchical Layout instead of force-directed)

### 2.3 MODIFYING RATE LAWS FOR THE OTHER REACTIONS (WHOSE EQUATIONS DID NOT NEED TO BE CHANGED, BUT HAD CUSTOM RATE LAWS)

**Step 10: GPI (**G6P=F6P)

(((Vbpgi*Kpgig6p/Kpgif6p*0.45)*G6P/Kpgig6p)-(Vbpgi*F6P/Kpgif6p))/(1+F6P/Kpgif6p + G6P/Kpgig6p)

Keyword: Phosphoglucoisomerase; Organism: Oryctolagus cuniculus

EC no: 5.3.1.9

Both K_m_ values are available on BRENDA

Kpgif6p = 0.01 [21]

Kpgig6p = 0.03 [21]

**Step 11: ALDOA** (FBP=DHAP+GAP)

((Vfald*FBP/KaldFBP)- ((Vfald*Kaldgap*Kalddhap/(KaldFBP*0.000095))*DHAP*GAP/(Kaldgap*Kalddhap)))/(1+FBP/KaldFBP+GAP/Kaldgap + DHAP/Kalddhap)

Keyword: Aldolase; Organism: Oryctolagus cuniculus

EC=4.1.2.13

All three K_m_ values are available on BRENDA.

KaldFBP = 0.06 [22]

Kaldgap = 1 [22]

Kalddhap = 2 [22]

**Step 12: TPI1** (GAP=DHAP)

((Vftpi*GAP/Ktpigap)-((Vftpi*Ktpidhap/(Ktpigap*19.2))*DHAP/Ktpidhap))/(1 + GAP/Ktpigap + DHAP/Ktpidhap)

Keyword: triose-phosphate isomerase; Organism: Oryctolagus cuniculus

E.C. 5.3.1.1

Both K_m ­_values are available on BRENDA

Ktpigap = 0.42 [23]

Ktpidhap = 0.75 [23]

**Step 13: PGAM4** (P2G=P3G)

((Vfpgm*P3G/Kpgm3pg)-((Vfpgm*Kpgm2pg/(Kpgm3pg*0.18))*P2G/Kpgm2pg))/(1+P3G/Kpgm3pg + P2G/Kpgm2pg)

Keyword: Phosphoglycerate Mutase; Organism: Homo sapiens

EC: 5.4.2.11

From BRENDA

Kpgm3pg = 0.4 [24]

No value of Kpm2pg for homo sapiens on BRENDA. That said, there are very few results available with P2G as the substrate.

From paper: 0.014 (chicken) [25]

**Step 14: ENO1** (P2G=PEP)

((Vfen*P2G/Ken2pg)-((Vfen*Kenpep/(Ken2pg*0.49))*PEP/Kenpep))/(1+P2G/Ken2pg + PEP/Kenpep)

Keyword: Enolase; Organism: Oryctolagus cuniculus

EC no: 4.2.1.11

Ken2pg = 0.061 [26]

Kenpep = 0.25 [26]

## 3- Reactions for Drug Treatment with Vemurafenib or Dabrafenib

### 3.1 Treatment with Vemurafenib

The following reaction was added to ERK_Akt_Wnt_SBML-BRaf.cps to construct the Vemurafenib treated version ERK_Akt_Wnt_SBML-BRaf-Vem.cps

$$pBRaf\to BRaf;Vem (1)$$

Rate Law: Modified Michaelis-Menten

$$\frac{Kcat*modifier*substrate}{km+substrate} (2)$$

Kcat = 1/s

Km = 100 nM

[Vem]_0 = 1000 nM

### 3.2 Treatment with Dabrafenib

The following reactions were added to ERK_Akt_Wnt_SBML-BRaf.cps to construct the Dabrafenib treated version ERK_Akt_Wnt_SBML-BRaf-DFB.cps

Note: Mass action (reversible) is selected as the rate law for all the reactions below.

$$BRaf+DBF\underset{d_{4}}{\underset{\rightleftharpoons}{a_{4}}} BRaf.DBF$$

$$BRaf.MEK+DBF\underset{d_{4}}{\underset{\rightleftharpoons}{a_{4}}} BRaf.MEK.DBF$$

$$BRaf.pMEK+DBF\underset{d_{4}}{\underset{\rightleftharpoons}{a_{4}}} BRaf.pMEK.DBF$$

$$BRaf.DFB\underset{d_{2}}{\underset{\rightleftharpoons}{a_{2}}} BRaf.MEK.DBF$$

$$BRaf.DBF+pMEK\underset{d_{2}}{\underset{\rightleftharpoons}{a_{2}}} BRaf.pMEK.DFB$$

| Constant | Value | Reference |
| --- | --- | --- |
| a_2_ | 0.106 µM^-1^s^-1^ | [27] |
| d_2_ | 0.02385 s^-1^ | [28] |
| a_4_ | 0.106 µM^-1^s^-1^ | [27] |
| d_4_ | 0.0000593 s^-1^ | [28] |

**Table S1**: Kinetic Constants used for Dabrafenib-related reactions

- Detailed steps for running comparative simulations and parameter perturbations can be found in CYTOCOPASI APP MANUAL – Sections 8.3-4

1. Fuentes, M.E., et al., *Kinetics of intra‐and intermolecular zymogen activation with formation of an enzyme–zymogen complex.* The FEBS Journal, 2005. **272**(1): p. 85-96.

2. Lambeth, M.J. and M.J. Kushmerick, *A computational model for glycogenolysis in skeletal muscle.* Annals of biomedical engineering, 2002. **30**(6): p. 808.

3. DAUGHERTY, J.P., W.F. KRAEMER, and J.G. JOSHI, *Purification and properties of phosphoglucomutase from Fleischmann's yeast.* European journal of biochemistry, 1975. **57**(1): p. 115-126.

4. Vereb, G., A. Fodor, and G. Bot, *Kinetic characterization of rabbit skeletal muscle phosphorylase ab hybrid.* Biochimica et Biophysica Acta (BBA)-Protein Structure and Molecular Enzymology, 1987. **915**(1): p. 19-27.

5. Tanabe, S., M. Kobayashi, and K. Matsuda, *Yeast glycogen phosphorylase: kinetic properties compared with muscle and potato enzymes.* Agricultural and biological chemistry, 1988. **52**(3): p. 757-764.

6. Madsen, N.B., *The inhibition of glycogen phosphorylase by uridine diphosphate glucose.* Biochemical and Biophysical Research Communications, 1961. **6**(4): p. 310-313.

7. Ariki, M. and T. Fukui, *Inhibition of α-glucan phosphorylase by α-D-glucopyranosyl fluoride.* The Journal of Biochemistry, 1975. **78**(6): p. 1191-1199.

8. Oikonomakos, N.G., et al., *Binding of N‐acetyl‐N′‐β‐d‐glucopyranosyl urea and N‐benzoyl‐N′‐β‐d‐glucopyranosyl urea to glycogen phosphorylase b: Kinetic and crystallographic studies.* European journal of biochemistry, 2002. **269**(6): p. 1684-1696.

9. Chrysina, E.D., et al., *Binding of β-D-glucopyranosyl bismethoxyphosphoramidate to glycogen phosphorylase b: Kinetic and crystallographic studies.* Bioorganic & medicinal chemistry, 2005. **13**(3): p. 765-772.

10. Gold, A.M., R.M. Johnson, and J.K. Tseng, *Kinetic mechanism of rabbit muscle glycogen phosphorylase a.* Journal of Biological Chemistry, 1970. **245**(10): p. 2564-2572.

11. Nagata, K., K. Suzuki, and K. Imahori, *Analysis of the allosteric properties of rabbit muscle phosphofructokinase by means of affinity labeling with a reactive ATP analog.* The Journal of Biochemistry, 1979. **86**(5): p. 1179-1189.

12. Merry, S. and H.G. Britton, *The mechanism of rabbit muscle phosphofructokinase at pH8.* Biochemical Journal, 1985. **226**(1): p. 13-28.

13. LAMBEIR, A.M., et al., *The cytosolic and glycosomal glyceraldehyde‐3‐phosphate dehydrogenase from Trypanosoma brucei: Kinetic properties and comparison with homologous enzymes.* European journal of biochemistry, 1991. **198**(2): p. 429-435.

14. Furfine, C.S. and S.F. Velick, *The acyl-enzyme intermediate and the kinetic mechanism of the glyceraldehyde 3-phosphate dehydrogenase reaction.* Journal of Biological Chemistry, 1965. **240**(2): p. 844-855.

15. Szabó, J., et al., *Communication between the nucleotide site and the main molecular hinge of 3-phosphoglycerate kinase.* Biochemistry, 2008. **47**(26): p. 6735-6744.

16. Boehme, C., et al., *Chemical and enzymatic characterization of recombinant rabbit muscle pyruvate kinase.* Biological Chemistry, 2013. **394**(5): p. 695-701.

17. Dyson, R.D., J.M. Cardenas, and R.J. Barsotti, *The reversibility of skeletal muscle pyruvate kinase and an assessment of its capacity to support glyconeogenesis.* Journal of Biological Chemistry, 1975. **250**(9): p. 3316-3321.

18. Pettit, S.M., D.A. Nealon, and A.R. Henderson, *Purification of lactate dehydrogenase isoenzyme-5 from human liver.* Clinical chemistry, 1981. **27**(1): p. 88-93.

19. Talaiezadeh, A., et al., *Kinetic characterization of lactate dehydrogenase in normal and malignant human breast tissues.* Cancer cell international, 2015. **15**(1): p. 1-9.

20. Sempere, S., A. Cortes, and J. Bozal, *Kinetic mechanism of guinea-pig skeletal muscle lactate dehydrogenase (M4) with oxaloacetate-NADH and pyruvate-NADH as substrates.* International Journal of Biochemistry, 1981. **13**(6): p. 727-731.

21. Noltmann, E.A., *9 Aldose-ketose isomerases*, in *The enzymes*. 1972, Elsevier. p. 271-354.

22. Morse, D.E. and B. Horecker, *The mechanism of action of aldolases.* Advances in enzymology and related areas of molecular biology, 1968. **31**: p. 125-181.

23. Krietsch, W., *[93] Triosephosphate isomerase from rabbit liver*, in *Methods in enzymology*. 1975, Elsevier. p. 438-442.

24. de Atauri, P., et al., *Characterization of the first described mutation of human red blood cell phosphoglycerate mutase.* Biochimica et Biophysica Acta (BBA)-Molecular Basis of Disease, 2005. **1740**(3): p. 403-410.

25. Rose, Z.B. and S. Dube, *Phosphoglycerate mutase. Kinetics and effects of salts on the mutase and bisphosphoglycerate phosphatase activities of the enzyme from chicken breast muscle.* Journal of Biological Chemistry, 1978. **253**(23): p. 8583-8592.

26. Pietkiewicz, J., et al., *Inhibition of human muscle-specific enolase by methylglyoxal and irreversible formation of advanced glycation end products.* Journal of Enzyme Inhibition and Medicinal Chemistry, 2009. **24**(2): p. 356-364.

27. Hamis, S.J., et al., *Quantifying ERK activity in response to inhibition of the BRAFV600E-MEK-ERK cascade using mathematical modelling.* British Journal of Cancer, 2021. **125**(11): p. 1552-1560.

28. VanScyoc, W.S., et al., *Enzyme kinetics and binding studies on inhibitors of MEK protein kinase.* Biochemistry, 2008. **47**(17): p. 5017-5027.
